# Integrative modelling of TIR domain-containing adaptor molecule inducing interferon-β (TRIF) provides insights into its autoinhibited state

**DOI:** 10.1186/s13062-017-0179-0

**Published:** 2017-04-20

**Authors:** Jarjapu Mahita, Ramanathan Sowdhamini

**Affiliations:** National Centre for Biological Sciences, Tata Institute of Fundamental Research, GKVK Campus, Bellary Road, Bangalore, 560065 India

**Keywords:** TRIF, Autoinhibition, Docking, Molecular dynamics simulations, Mutual information, Residue network analysis

## Abstract

**Background:**

TRIF is a key protein in antiviral innate immunity, operating downstream of TLRs. TRIF activation leads to the production of interferon-β and pro-inflammatory cytokines. There is evidence from experiments to suggest that the N-terminal domain of TRIF binds to its TIR domain to avoid constitutive activation. However, no structure of a complex between the N-terminal domain and the TIR domain exists till date. The disordered nature of the region connecting the N-terminal domain and the TIR domain compounds the issue of elucidating the mechanism of autoinhibition of TRIF. In this study, we have employed an integrative approach consisting of mutual information analysis, docking, molecular dynamics simulations and residue network analysis, in combination with existing experimental data to provide a glimpse of TRIF in its autoinhibited state.

**Results:**

Our extensive docking approach reveals that the N-terminal domain binds to the BB loop-B helix region of the TIR domain, consistent with experimental observations. Long length molecular dynamics simulations of 1 microsecond performed on the docked model highlights residues participating in hydrogen bonding and hydrophobic interactions at the interface. A pair of residues present in the vicinity of the interface is also predicted by mutual information analysis, to co-evolve. Residues mediating long-range interactions within the TIR domain of TRIF were identified using residue network analysis.

**Conclusions:**

Based on the results of the modelling and residue network analysis, we propose that the N-terminal domain binds to the BB loop region of the TIR domain, thereby preventing its homodimersation. The binding of TRIF to TLR3 or TRAM could induce a slight conformational change, causing the interactions between the N-terminal domain and TIR domain to disrupt, thereby exposing the BB loop and rendering it amenable for higher-order oligomerisation.

**Reviewers:**

This article was reviewed by Michael Gromiha, Srikrishna Subramaniam and Peter Bond (nominated by Chandra Verma).

**Electronic supplementary material:**

The online version of this article (doi:10.1186/s13062-017-0179-0) contains supplementary material, which is available to authorized users.

## Background

The Toll/Interleukin-1 receptor-like (TIR) domain-containing adaptor molecule-1 (TICAM-1) or TIR domain-containing adaptor molecule inducing interferon-β (TRIF) belongs to the Toll-like receptor (TLR) family of pathogen-recognition receptors (PRRs) and associated downstream adaptor proteins [1, 2]. These proteins, which possess a common TIR domain, play a critical role in the innate immune system through the Toll-like receptor-mediated detection of pathogen-associated molecular patterns (PAMPS) from microorganisms and damage-associated molecular patterns (DAMPS) in vertebrates [3, 4]. Detection by the receptors triggers a series of steps involving recruitment of the downstream adaptor proteins to the receptors resulting in a signalling cascade. The end product of this signalling cascade is the activation of transcription factors like NF-kB (nuclear factor kappa-light chain enhancer of activated B cells) and IRFs (interferon-regulating factors) leading to production of pro-inflammatory cytokines and interferons. Subsequently, this leads to stimulation of the adaptive immune system in vertebrates [5].

The other TIR domain-containing adaptor proteins include MyD88 (Myeloid differentiation primary response gene 88), MAL/TIRAP (MyD88 adaptor-like/TIR domain-containing adaptor protein), TRIF/TICAM-1 (TIR domain-containing adaptor molecule-inducing interferon-β/TIR domain-containing molecule 1), TRAM/TICAM-2 (TRIF-related adaptor molecule/TIR domain-containing adaptor molecule 2) and SARM (sterile-α and HEAT-Armadillo motifs containing protein). MyD88 is utilized by all the receptors except Toll-like receptor 3 (TLR3) while TRIF is involved in TLR3 and Toll-like receptor 4 (TLR4) signalling [6, 7]. Emerging evidence suggests that TRIF might be involved in Toll-like receptor 2 (TLR2) signalling too [8, 9]. The TIR domain responsible for mediating the protein-protein interactions between the TLRs and adaptor proteins, as well as amongst the adaptor proteins has a core structure of a flavodoxin-like fold, containing alternating α-helices and β-strands. The number of strands could vary between four and five and there are variations in the length of connecting loops in different domains within this family [10]. Among these loops, the BB loop holds remarkable functional significance through its requirement for oligomerisation of TLR receptors and adaptor proteins into signalsomes. The conserved proline residue in the BB loop has been shown to be critical to the integrity of the TLR signalsome [11, 12].

**Fig. 1:**
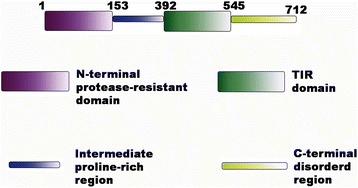
Domain architecture of TRIF. The full-length TRIF protein is 712 amino acids. The N-terminal protease resistant domain spans residues 1 to 153, residues 154–392 make up the intermediate proline-rich disordered region, residues 393–545 constitute the TIR domain while the C-terminal disordered region comprises of residues 546–712

The TRIF protein is 712 amino acids long, comprising of an N-terminal protease resistant domain, an intermediate long disordered proline-rich region, a TIR domain and a C-terminal disordered region (containing a RIP homotypic interaction motif (RHIM) domain) (Fig. 1). The N-terminal domain has a structure similar to the IFIT (interferon-induced proteins with tetracotripeptide repeats) family of proteins [13, 14]. The disordered region between the N-terminal domain and the TIR domain contains binding sites for many downstream proteins like TBK1 (TANK-binding kinase 1), and TRAFs (Tumour necrosis factor (TNF) receptor associated factor (TRAF2 and TRAF6) [15]. Depending on the proteins that bind to it, TRIF can mediate both NF-kB and IRF-3(interferon regulatory factor-3) activation. While TRIF requires the TRAM adaptor to bind to TLR4, it can directly bind to TLR3 [16]. TLR4 recognises bacterial lipopolysaccharide and is localised at both the plasma membrane and the endosome while TLR3 senses viral double stranded RNA and has been observed to be localised mainly in intracellular compartments, notably the endosome [17, 18]. Findings from multiple research groups have established that TRIF is expressed in low levels and remains diffused in the cytoplasm of resting cells. Upon TLR3 stimulation by poly I:C, a synthetic analogue of viral double stranded RNA, TRIF localises to the endosome where it makes a transient interaction with the receptor before dissociating to form speckle-like structures in the cytoplasm [19]. Overexpressed TRIF has also been observed to bind constitutively with inactive TLR3 in resting HEK293 cells [20].

Homodimerisation of TRIF is essential for it to function smoothly [21]. The Proline 434 residue of the BB-loop of the TIR domain of TRIF is indispensable for homodimerisation of TRIF. However, the monomeric form of TRIF is sufficient for binding to the dimeric form of the TLR3 receptor [21]. It has been observed, on separate occasions, that the N-terminal domain of TRIF binds to its TIR domain because a mutant construct lacking the N-terminal domain showed higher promoter activation relative to the wild type [13, 22]. This has led to the view that the N-terminal domain binds to the TIR domain as a mechanism of autoinhibition. The binding of the TIR domain to TLR3 might disrupt the interaction of the former with the N-terminal domain and expose an additional surface of the TIR domain as well as the intermediate proline-rich region. This increase in surface area, due to exposure of the latter, could then provide a basis for the interaction partners to bind to it. However, so far, no structure of a complex between the N-terminal domain and the TIR domain has been solved. The disordered nature of the region connecting both these domains has also hampered investigations into the mechanism of its autoinhibition. Here, we have used an approach consisting of integrative modelling, docking and molecular dynamics simulations to present a putative structure of the N-terminal domain in complex with the TIR domain.

## Results and discussion

### Co-evolving residues between the N-terminal protease-resistant domain and the TIR domain

The protease-resistant N-terminal domain of TRIF is 153 residues long, while the TIR domain consists of 154 residues. Given that the binding of the N-terminal domain onto the TIR domain seems crucial in order to avoid its constitutive activation, we reasoned that it could be an evolutionary conserved mechanism, at least in vertebrates. Since interacting residues at the interface are more likely to co-evolve in order to maintain the integrity of the complex, we wanted to predict which pairs of residues between the two domains are most likely to co-evolve. Many studies have utilised information from amino acid co-evolution to identify residues that mediate protein-protein interactions. Most of these methods require a large number of sequences, usually 500 or more. However, for less number of sequences, mutual information analysis is considered suitable. We performed mutual information analysis to predict co-evolving pairs of residues between the N-terminal domain and the TIR domain. The method uses information theory to calculate occurrences of amino acid pairs. The CMAT (Correlated Mutation Analysis Tool) [23] algorithm considers information contained in a sequence alignment to calculate the joint probability of the occurrence of an amino acid pair. In addition, it has also incorporated terms corrected for the background noise due to phylogeny and random noise. The various terms used in prediction of co-evolving residues are as below.

$$ {P}_{P P}\left({x}_i,{y}_j\right)=\left(1-\tau \right){P}_{obs}\left({x}_i,{y}_j\right)+\tau q\left({x}_i\right) q\left({y}_j\right) $$

*q(x*_*i*_ *) :probability of amino acid x being present at position i*

*q(y*_*j*_ *):probability of amino acid y being present at position j*

*P*_*PP*_(*x*_*i*_, *y*_*j*_)*: Joint probability taking profile-based pseudocount into account*

*P*_*obs*_(*x*_*i*_, *y*_*j*_)*: Joint probability without pseudocount*

$$ \tau =\frac{1+ b}{e^{aNeff\left( i, j\right)}+ b} $$

*N*_*eff*_ *(i,j): Effective number of sequences aligned at both positions i and j*

*a,b: positive constants*

$$ M I\left( i, j\right)={\displaystyle \sum_x{\displaystyle \sum_y P\left({x}_i,{y}_j\right) \log \left(\frac{P\left({x}_i,{y}_j\right)}{P\left({x}_i\right) P\left({y}_j\right)}\right)}} $$

$$ P\left({x}_i\right)={\displaystyle \sum_y P\left({x}_i,{y}_j\right)} $$

$$ P\left({y}_i\right)={\displaystyle \sum_x P\left({x}_i,{y}_j\right)} $$

*P(x*_*i*_ *, y*_*j*_ *): Joint probability of amino acid x being at position i and amino acid y being at position j.*

*MI*_*p*_ *(i,j) = MI(i,j) – MI(i,* ***·*** *)MI(* ***·*** *,j)MI(* ***·*** *,* ***·*** *)*

*MI(i,* ***·*** *): MI of i averaged over all other positions in the alignment*

*MI(* ***·*** *,j): MI of j averaged over all other positions in the alignment*

*MI(* ***·*** *,* ***·*** *): MI averaged over all positions*

A total of 97 sequences, corresponding to the N-terminal domain and the TIR domain of TRIF, were collected from different organisms. The list of these organisms is listed in Additional file 1: Appendix S1. The resulting concatenated multiple sequence alignment is shown in Additional file 2: Figure S1. The amino acid residue pairs, predicted by CMAT to co-evolve on the basis of their MIp and MIc scores, are shown in Additional file 3: Table S1. Out of the total of 24 pairs predicted, seven pairs of residues correspond to intra-domain residue pairs (within the N-terminal domain), nine intra-domain residue pairs (from within the TIR domain) and eight inter-domain residue pairs. However, these results must be interpreted with caution due to the slightly lower number of sequences considered.

### Putative binding mode between N-terminal domain and TIR domain

**Fig. 2:**
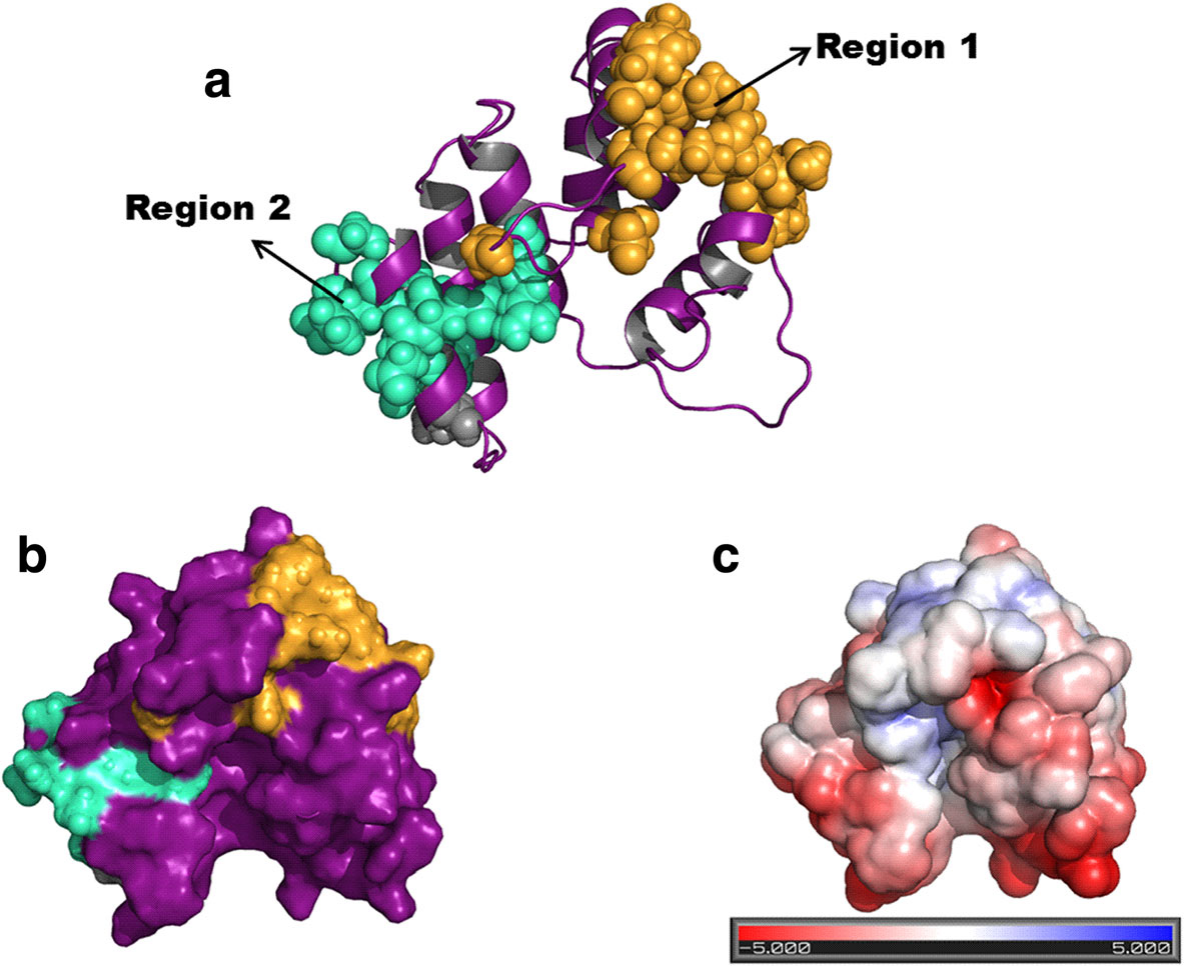
**a** Cartoon representation of the N-terminal protease-resistant domain. The residues making up Region 1 and Region 2 (see text for details) are highlighted as light brown and cyan coloured spheres respectively. **b** Surface representation with the same colour coding as in **a** and **c** APBS-generated electrostatic surface potential representation showing the presence of the major cleft between these two regions. All images were created using PyMOL visualisation software

The structures of the TIR domain of TRIF (PDB ID: 2M1X) and the N-terminal protease-resistant domain (PDB ID: 4BSX) were used for structural analysis and docking [24, 25]. The residues corresponding to the BB loop (residues 428–439) and the αB helix (residues 441–452) have been demonstrated to be essential for its binding to TRAM and TLR4 [26] (Additional file 4: Figure S2a). Similarly, another study has also elucidated the importance of the BB loop in homodimerisation of TRIF and the residues Gln 518, Ile 519, Arg 521 and Lys 522 (RK site) in mediating heterotypic interactions with TRAM [25]. This electropositive patch is located on the surface opposite to the BB loop region (Additional file 4: Figure S2b). The N-terminal domain contains tetracotripeptide repeats which show structural similarity to the TPRs located in the N-terminal region of IFIT proteins [24]. Inspection of its surface reveals presence of two clefts. Residues lining the major cleft are mostly acidic, but the residues within the cleft are mainly non-polar residues. Binding site prediction algorithms, meta-PPISP [27] and SPPIDER [28], pinpoint certain sets of residues which cluster into two distinct regions on the N-terminal domain. These regions are located opposite to each other. One region (denoted as Region I) includes the residues Leu 24, Lys 27, His 28, Lys 31, Gly 36, Asp 41, Lys 50, Leu 51, Asn 53, Thr 55, Glu 56 and Arg 58. Residues Arg 98, Gln 119, Gln 120, Val 122, Gln 137, Asp 138, Glu 139, Arg 141, Gly 145 and Asp 147 make up Region 2. When mapped onto a surface representation of the N-terminal domain, it is observed that Region 1 and Region 2 are located on either side of the major cleft (Fig. 2). A multiple sequence alignment of around 100 N-terminal domain sequences from mammals, birds and fish further reveal that the residues conserved among these organisms are mostly non-polar residues. Interestingly, most of these conserved residues cluster either in Region 1 or Region 2 (Additional file 5: Figure S3).

A model of the interaction between N-terminal domain and the TIR domain of TRIF was constructed through multiple docking schemes wherein different regions of each domain was used to guide docking. Analysis of all the top poses showed that the BB loop was indeed present in all the interfaces. A summary of the residues predicted to be in the interface by Cluspro for each case is listed in Additional file 6: Table S2. Interestingly, even after specifying the 522R/523 K site on the TIR domain to guide its docking to the acidic residues (Region 1) lining the major cleft of the N-terminal domain, the top poses returned by Cluspro were those in which the BB loop was present at the interface. The refined docked complex obtained by subsequently carrying out restrained docking using HADDOCK [29] will henceforth be referred to as N-TIR complex (Additional file 7: Figure S4).

### Molecular dynamics simulations reveal stable nature of interactions present at the interface between N-terminal domain and TIR domain in the docked N-TIR complex

**Fig. 3:**
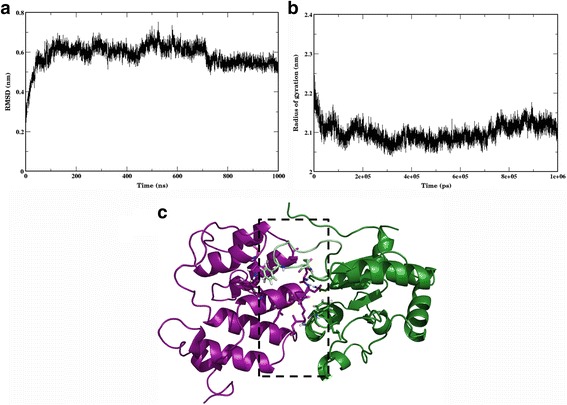
**a** A plot of RMSD of backbone atoms of the N-TIR complex (for model coordinates, please see Additional file 13) along the trajectory, using the initial equilibrated structure generated post NPT equilibration as a reference. **b** A plot showing the radius of gyration of the N-TIR complex along the MD trajectory. **c** The three-dimensional model of the complex formed by the N-terminal protease-resistant domain (*marked in purple* in Fig. 1) and the TIR domain (*marked in green* in Fig. 1) extracted from the equilibrated portion of the MD trajectory

**Fig. 4:**
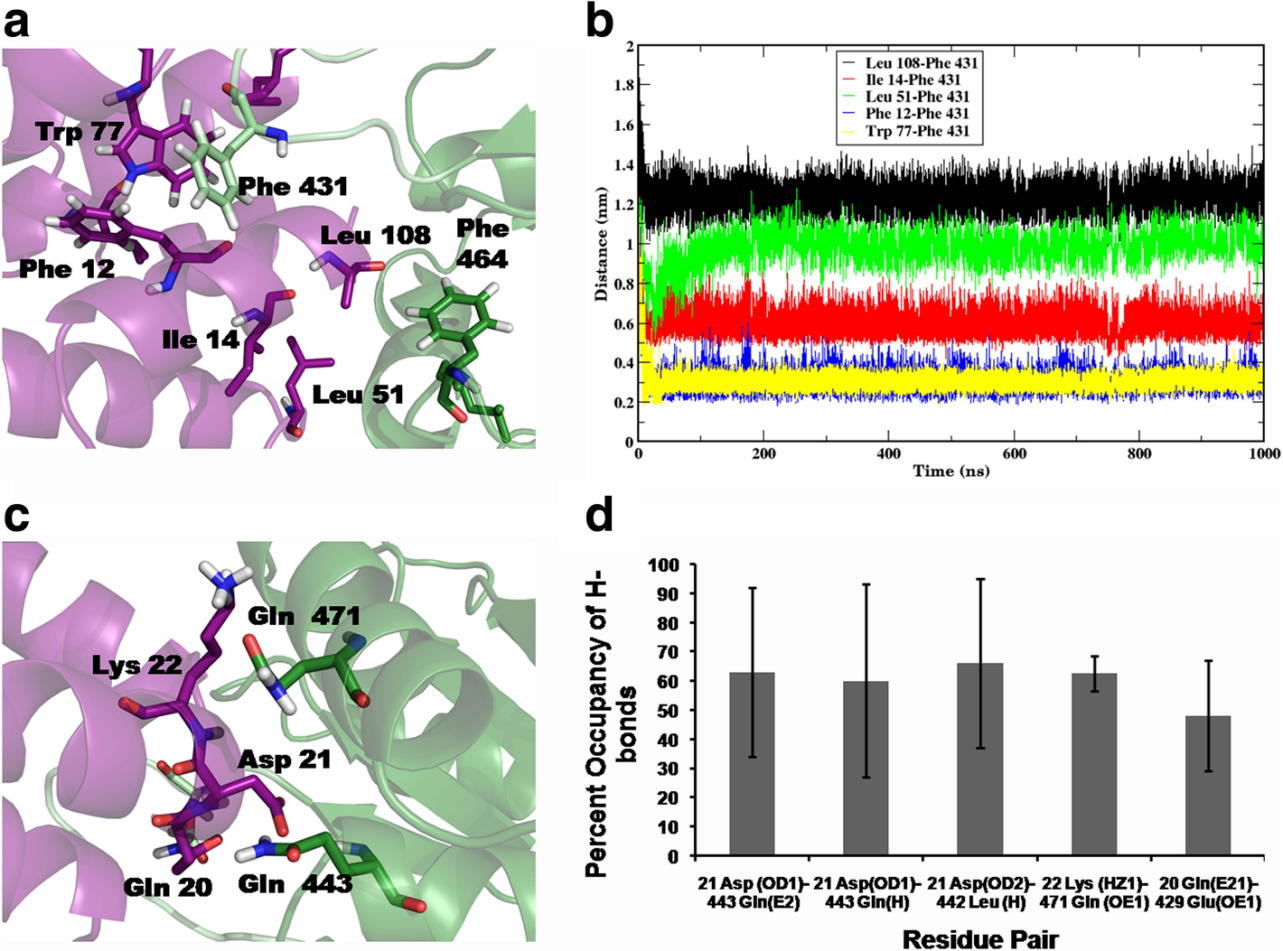
**a** Zoom-in of the interface showing hydrophobic interactions, with the residues of interest being represented as sticks. The residues Phe 12 and Trp 77 on the N-terminal domain (*purple*) are located in close proximity to Phe 431 of the TIR domain (*green*). **b** A plot of minimum distance between select residue pairs: Leu 108-Phe 431(*black*), Ile 14-Phe 431(*red*), Leu 51-Phe 431(*green*),Phe 12-Phe 431 (*blue*) and Trp 77-Phe 431(*yellow*) throughout the time course of the simulation. Phe431 of the TIR domain appears to play an important role in the interaction of the domains. **c** Residues involved in the formation of stable inter-domain hydrogen bonds. The residues Gln 20, Asp 21, Lys 22 of the N-terminal domain (*purple*) and residues Gln 443, Gln 471 of the TIR domain (*green*) are represented as sticks. **d** Percent hydrogen bond occupancy between the different donor-acceptor residue pairs as shown in **c**


Three independent molecular dynamics simulations, to a timescale of 1 microsecond each, were performed on the N-TIR complex to assess its stability (Additional file 8: Video S1). The variation in the backbone RMSD and the radius of gyration along the trajectory are shown in Fig. 3a and b, respectively. It is evident that the complex remains stable throughout the length of the simulations (Additional file 9: Figures S5a-c). The model structure of the N-TIR complex extracted from the MD trajectory, at the end of 1 microseccond, is shown in Fig. 3c. The interactions operating at the interface are composed mainly of hydrophobic interactions and hydrogen bonds. Potential hotspot residues identified by our in-house program, PPCheck [30], include Phe 12, Gln 20, Asp 21, Leu 24, Tyr 25, Trp 77 on the N-terminal domain and Phe 431, Ser 440, Cys 441, Leu 442, Gln 443 on the TIR domain. Phe 431 is located in the BB loop, while the residues Ser 440-Gln 443 constitutes the B helix. This is consistent with their functional role in mediating protein-protein interactions as elucidated from mutagenesis studies [26]. Residues Phe 12, Leu 51, Trp 77 and Leu 108, on the N-terminal domain are engaged in hydrophobic interactions with Phe 431 on the TIR domain. The close proximity of the phenyl ring and indole ring of Phe 12 and Trp 77, respectively, to the phenyl ring of Phe 431 denotes energetically-favourable pi-pi stacking interactions occurring between these residues (Fig. 4a). The distance between Phe 431 and the surrounding non-polar residues Phe 12, Ile 14, Leu 51, Trp 77 and Leu 108 was monitored throughout the simulations and is shown as a plot in Fig. 4b. The number and stability of the hydrogen bonds at the interface could reflect the stable nature of the complex. Hydrogen bonds are formed between amino acid residue pairs, Asp 21-Gln 468, Asp 21-Gln 443, Asp 21-Leu 442, Lys 22-Gln 471 and Gln 20-Glu 429 (Fig. 4c and Additional file 10: Figure S6(a–d)). Hydrogen bonds having occupancy (the ratio of the number of times that particular H-bond is present relative to the total time of the simulation) more than 50% were considered as stable hydrogen bonds. The occupancy of each hydrogen bond pair is shown in Fig. 4d. Additionally, snapshots were extracted, every 1 ns from the equilibrated portion of the trajectory (from 100 ns onwards), and analysed for various interface energies using the PPCheck algorithm [30] The plots of these different energy values versus time are shown in Additional file 11: Figure S7(a–f). The hydrogen bond energies range from 0 kcal/mol to −30 kcal/mol, while the van der Waals energies, at the interface, range around −200 to −250 kcal/mol. The average number of residues present at the interface is observed to be around 90 and the values of the normalized energy per residue falls between −2 and −3 kcal/mol. Together, these results suggest that the model of N-TIR domain complex we have constructed is energetically stable. Indeed, this could resemble the interactions formed under physiological conditions between the two domains and is also in line with the experimental observation that the N-terminal domain folds on to the N-terminal part of the TIR domain [22]. Furthermore, the predicted co-evolving residues were mapped onto one of the snapshots extracted from the trajectory (Additional file 12: Figure S8). While the inter-domain co-evolving residue pairs do not directly interact at the interface, in some of these inter-domain residue pairs, one of the residues making up the residue pair, is present at the interface.

Additional file 8: Video S1.Molecular dynamics simulations of the TRIF N-TIR docked complex. The N-terminal domain is coloured in violet while the TIR domain in green. Residues of the N-terminal domain participating in either H-bonding or hydrophobic interactions are represented as blue sticks while those from the TIR domain are represented as red sticks. (MPG 108645 kb)


### Shortest path of communication between the BB loop and the RK site on the TIR domain of TRIF

**Fig. 5:**
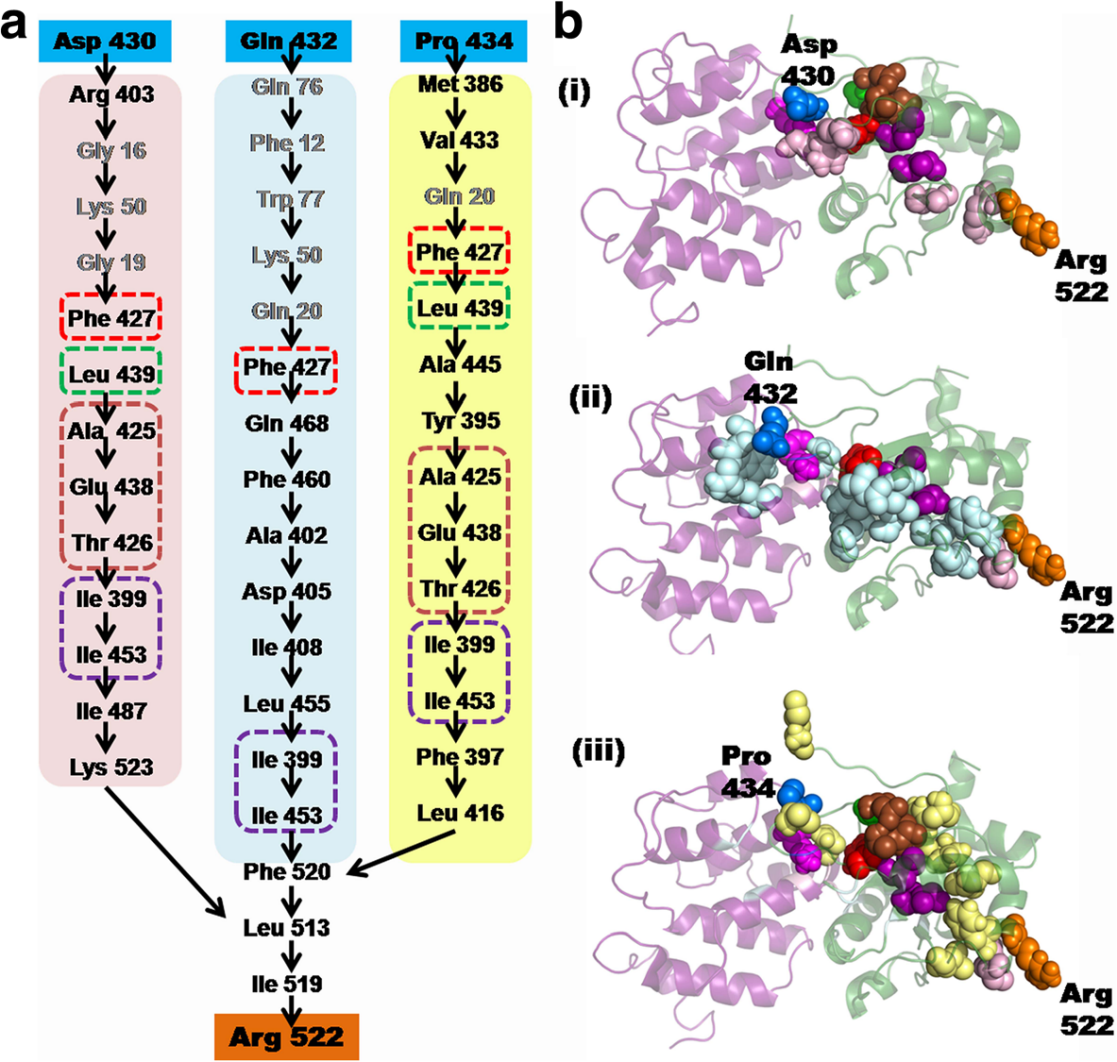
**a** Residues identified by the PSN-Ensemble program to mediate long-distance interaction between select residues of the BB loop (Pro 434, Gln 432 and Asp 430, *blue boxes*) and Arg 522 (*orange box*) in the RK site. It is seen that of the three chosen BB loop residues, the shortest path i.e, the path containing the least number of residues required to mediate interactions, is between Asp 430 and Arg 522. Residues that are common to at least 2 paths of communication are enclosed within coloured boxes. All three paths pass through the residue Phe 437 (*red box*) as well as residues Ile 399 and Ile 453 (*marked within purple box*). Residue Leu 439 (*green box*) and residues Ala 425, Glu 438, Thr 436 (*marked within brown box*) mediate long-distance communication between Asp 430 and Arg 522 as well as between Pro 434 and Arg 522. **b** Mapping of the path of communication between the source residues (*shown as blue spheres*) and sink residue Arg 522(*orange spheres*) onto the structure of the N-TIR docked complex. (i)Asp 430-Arg 522, (ii) Gln 432-Arg 522 and (iii) Pro 434-Arg 522. The intermediate residues connecting Asp 430 and Arg 522 are shown as pink spheres, those connecting Gln 432 and Arg 522 in light blue spheres while those connecting Pro 434 and Arg 522 are coloured *yellow*. Residues, that have been marked within the coloured boxes in (**a**), are highlighted by the same colours on the structure

Since the BB loop and 522R/523 K site are known to be important for the function of TRIF [25] and might act in a concerted manner, and given that they are located on opposite sides of the TIR domain, we sought to explore the network of residues that mediate the long range interaction between these two sites. Protein structure networks have emerged as a valuable means of identifying important residues crucial for the stability of the protein fold, as well as the residues involved in weak and strong non-covalent interactions [31–33]. We used the PSN-Ensemble program [34] to identify the shortest path of communication between select residues on the BB loop and the RK site from the MD snapshots. The residues Asp 430, Gln 432 and Pro 434, all part of the BB loop, were selected as the source residues and the residue Arg 522 as the sink residue. The residues that mediate the long-range communication between the source and sink residues are illustrated in Fig. 5a, while their relative position on the N-TIR complex is shown in Fig. 5b. Certain residues on the N-terminal domain (highlighted in grey in Fig. 5a) are also found to mediate these long-distance communications. These residues are located either directly at or in the vicinity of the N-TIR domain-domain interface. While there are differences in the residues that constitute each path of communication, all three paths pass through the residue Phe 427 on the TIR domain (Fig. 5). Moreover, the communication paths, Asp 430-Arg 522 and Pro 434-Arg 522, have multiple common residues (boxed in Fig. 5) suggesting that they form higher order residue networks. The average shortest path is observed between the residue Asp 430 and Arg 522. Remarkably, all the three paths converge at the residues Leu 513 and Ile 519. Apart from the 522R/523 K site, the 518Q/519I site has also been demonstrated to assist in the proper functioning of TRIF [25]. Besides, Leu 513 is also highly conserved across many species (Additional file 3: Figure S1).

Long-range interactions have also been studied in other proteins using residue network analysis. The number of residues mediating these long-range interactions is variable. For example, in the MetRS-tRNA complex, different communication paths were identified between the active site and the anticodon binding region and the average number of residues mediating the interactions was between 10 and 17 [35]. Hence, the shortest paths identified in our study are likely to make significant contributions towards mediating long-range interactions between the RK site and the BB loop. Indeed, such interactions could be important for signal transduction and serve as a starting point for further investigations into the mechanism of TRIF activation.

## Conclusions

Among the wide repertoire of proteins involved in innate immune signalling, TRIF is a key protein whose function is of major significance to the proper regulation of the innate immune system. The disordered proline-rich region between the N-terminal domain and the TIR domain is a major signalling hub for various downstream proteins leading to different signalling outcomes. Despite the critical role played by TRIF in antiviral innate immunity, the mechanism of its autoinhibition remains to be elucidated. It has been established that its N-terminal domain binds to the TIR domain to avoid constitutive activation, yet no structure of this complex is available. In this study, we have attempted to construct a feasible model of the interaction between N-terminal domain and the TIR domain of TRIF, by combining available experimental information with docking and molecular dynamics simulations studies. In our model, the N-terminal domain binds to the BB loop-B helix region of the TIR domain and this agrees with experimental observations reporting that the N-terminal domain binds to the N-terminal region of the TIR domain.

The results of our modelling and residue network analysis enables us to hypothesize that, upon TLR3 activation, the RK site on the TRIF TIR domain interacts with the TIR domain of TLR3. This RK site has also been shown to be required for binding to the TIR domain of TRAM [25]. This could induce a slight conformational change, disrupting the hydrophobic interactions and hydrogen bonds between the N-terminal domain and TIR domain through long-distance interactions (as shown in Fig. 5). This then exposes the BB loop on the TRIF TIR domain resulting in the ‘activated’ state of TRIF. Together, the BB loop and the C-terminal region contribute to the formation of a fully functional TRIF homodimer (or a higher-order oligomer), as a deletion of either the TIR domain or the C-terminal region resulted in decreased signalling (Additional file 14: Figure S9) [22]. A recent study by Vyncke and co-workers [36], in which the authors have delineated the arrangement of TIR domains in the Myddosome, also supports the hypothesis that the RK site on TRIF most likely binds to the TIR domain of TLR3.

MAVS is an adaptor protein associated with the innate immune receptor RIG-I (retinoic acid- inducible gene-like receptor-I) that also senses viral double stranded RNA, similar in function to TLR3, but localised in the cytoplasm instead. MAVS contains a N-terminal CARD domain for binding to the CARD domain of RIG-I (analogous in function to the TIR domain in TLRs), an intermediate disordered region and a C-terminal transmembrane domain for targeting it to the mitochondria [37]. Both MAVS and TRIF contain similar binding motifs in their intermediate disordered region and converge on the same downstream target [38]. Like TRIF, MAVS also remains in an autoinhibited conformation, which prevents it from spontaneous activation. Upon activation of RIG-I and binding of MAVS to it, prion-like MAVS filaments are formed [39, 40]. Additionally, the Ser residue of the pLxIS TBK1/IRF-3 binding motif located in the disordered region of these proteins is phosphorylated prior to IRF-3 binding [38]. An analysis of the disordered regions present in other proteins involved in antiviral innate immunity has reported that MAVS and TRIF contain the highest content of disorder among these categories of proteins [41]. Given that both MAVS and TRIF have very similar binding motifs and are present in an autoinhibited state, it is attractive to speculate that the autoinhibitory mechanism of TRIF could closely resemble that of MAVS. This possibility could be investigated through experiments and will help shed light on the exact mechanism of TRIF activation.

## Methods

### Predicting a model of the N-terminal domain bound the TIR domain as in the autoinhibited conformation of TRIF

#### Mutual information analysis

Mutual information was used to predict co-evolving residues between the N-terminal domain and the TIR domain of TRIF. Homologous sequences corresponding to these regions were extracted from both the Uniprot database and non-redundant database hosted at the National Centre for Biotechnology Information. Redundant sequences were removed and domain boundaries delineated using the Pfam domain database. Subsequently, sequences corresponding to a particular domain were aligned using MUSCLE and manually edited. Columns with >30% gaps were removed. The multiple sequence alignments of both domains were concatenated. Residue pairs that are predicted to co-evolve were identified using the CMAT (Correlated Mutations Analysis Tool) [23]. This algorithm extracts information contained in a sequence profile in the form of a pseudocount, namely profile-based pseudocount. This enables better accuracy of the estimated joint probability of a pair of amino acids x and y being present at positions i and j respectively, in a multiple sequence alignment.

#### Protein-protein docking

A series of dockings were attempted to identify a putative docked pose between the N-terminal domain and the TIR domain and to assess if the same docking pose was achieved through these different strategies and thus reduce the chances of false positives. The structure of N-terminal domain (PDB ID: 4BSX) and the representative NMR structure of the TIR domain (PDB ID: 2M1X) were used in this study. Prior to docking, the His 434 residue in the TIR domain was modified back to Proline and subsequently energy minimized in SYBYL. Two docking programs- Cluspro [42] and our in-house program, CAPSDOCK (Oommen K. Mathew and R. Sowdhamini, unpublished results), were used to generate docked poses. Different strategies were employed to generate the docked poses. Blind docking of the two domains was first performed using CAPSDOCK (Oommen K. Mathew and R. Sowdhamini, unpublished results) and the top models assessed for their interface residues. This was used as a starting point, coupled with known experimental information of the TIR domain and the conserved residues on the N-terminal domain, towards obtaining a more accurate model of the docked complex. Following this, multiple semi-guided docking runs were done using Cluspro [42]. A different region of each domain was used for guiding the docking. For example, using the BB loop residues of TIR domain to guide, it was predicted to bind to a certain region in the vicinity of the major cleft on the N-terminal domain. Similarly, using the acidic residues lining the major cleft of N-terminal domain to guide the docking, the top poses were those in which the BB loop was present in the interface Docked poses were ranked using DockScore [43] and interactions at the interface identified using PPCheck [30]. Both these softwares were developed in the lab. DockScore is a scoring scheme that utilizes various parameters such as the number of interfacial hydrophobic residues, spatial clustering of residues, residue conservation, number of short contacts and accessible surface area at the interface to discriminate between native and non-native poses. PPCheck calculates pseudoenergies for the various interactions operating at the protein-protein interface using distance thresholds to define a particular type of interaction. These pseudoenergies are used to quantify the strength of the protein-protein interactions. Additionally, PPCheck can also be used to identify hotspot residues. Subsequent to the analysis of the top ranked poses, further refinement was carried out by employing the HADDOCK docking program, using the N-terminal domain residues conserved in all vertebrates and the TIR domain BB loop residues to guide the docking, followed by ranking of the docked poses by DockScore, [43]. Interface residues were identified by PPCheck [30]. Electrostatic surface potential maps were generated through the APBS web server [44, 45].

#### Molecular dynamics simulations

To assess the stability of the docked pose, molecular dynamics simulations were carried out using the GROMACS 54a7 force field. The complex of the N-terminal domain and TIR domain was first processed and then solvated in a rhombic dodecahedron box using the SPCE water model. The distance between the edge of the box and the protein complex was set to 10 nm. The system was energy minimized using 50,000 steps of steepest descent with a time step of 1 femtosecond and no position restraints applied. The protein complex was then restrained while the solvent around it was equilibrated for 200 ps with a time step of 2 femtoseconds in order for the system to attain a temperature of 300 K. The V-rescale thermostat [46] was used for NVT equilibration. NPT equilibration was carried out using the Berendsen barostat [47] for 200 ps to set the pressure to 1 bar. Prior to production MD run, the restraints on the protein complex were released. Production MD was carried out for 1 microsecond using the leap-frog integrator with the time step set to 3 femtoseconds and the Parrinello-Rahman barostat [48] to maintain the pressure at 1 bar. To prevent the system from exploding due to a slightly higher timestep, certain hydrogen atoms were replaced by dummy atoms by use of the virtual sites option. The particle mesh-Ewald (PME) algorithm, with the Verlet cut-off scheme, was used for long-distance electrostatics [49]. The cut-off distance for long-distance electrostatics and van der Waals interactions was set to 1 nm. Bonds including heavy atom-H bonds were restrained using the LINCS algorithm [50]. Positions and velocities were saved every 3334 steps (10 ps). Hydrogen bonds were identified using the *g_hbond* tool implemented in GROMACS. The occupancy of each hydrogen bond was calculated by using a Python script, readHBmap.py, supplied for use with GROMACS trajectories. For analyses of interaction energies at the interface of the N-TIR complex, snapshots were extracted at every 1 ns, and provided as input to the PPCheck algorithm and the results of all snapshots were consolidated using short python scripts developed in the lab.

### Identification of paths of communication using protein structure networks

The possible path of communication between the residues of the BB loop and the RK site on the TIR domain of TRIF were investigated using PSN-Ensemble [34]. The program considers amino acids as nodes. The non-covalent interaction between two residues is specified by an interaction cut-off and edges are constructed between those nodes whose interaction strength is above the specified cut-off. The protein is therefore represented as a network of nodes connected by edges. Clusters are defined as group of nodes. Likewise, for every MD snapshot, the network is constructed. Dynamically stable networks are those that are present in a certain percentage of snapshots. The PSN-Ensemble program uses the Floyd-Warshal algorithm to calculate the shortest path between a pair of residues. In our analysis, first, a suitable cut-off of the interaction strength (I_min_ value) of 2.5 was chosen after plotting the size of the largest cluster versus different values of interaction strength. The value at which the cluster size undergoes a transition is taken as the I_min_ value. MD snapshots were extracted every 1 ns from the trajectory and the cut-off for dynamic stability was 50%. For each snapshot, the shortest path of communication between select residues on the BB loop and residue Arginine 522 of the RK site was determined. The average shortest path between a pair of source and sink residue was obtained by calculating the shortest path in each of the MD snapshots and taking their average.

## Reviewers’ comments

### Reviewer’s report 1: Michael Gromiha, Department of Biotechnology, Indian Institute of Technology Madras

In this work, the authors utilized computational methods and experimental data to understand the auto inhibited state of TRIF. They have performed extensive computations including mutational analysis, docking, molecular dynamics simulations and normal mode analysis. Using the results obtained from the study the authors proposed that the N-terminal domain binds to the BB loop region of the TIR domain and binding of TRIF induce conformational changes, which disrupt the interactions between the N-terminal and TIR domain thereby exposing the BB loop and rendering it amenable for higher-order oligomerisation. The work is interesting and exhaustive to derive the conclusions. The manuscript is well written and necessary details are provided.

1. Different docking algorithms are used in the manuscript (say Cluspro and capsdock etc.). The consensus binding sites obtained with these programs could be discussed.

Author response: *Thank you for the suggestions. We have now provided the consensus binding sites obtained with each program. They are listed in Table S2.*

2. Likewise several modelling software are used to predict the proline-rich region between the N-terminal and TIR domain of TRIF, which utilize different techniques. The reliable models may be discussed along with available experimental data.

Author response: *We have included additional details of the modelling softwares used to predict the model of the proline-rich region between the N-terminal domain and the TIR domain of TRIF, in this revised version of the manuscript.*

3. References should be properly formatted. Volume numbers are missing in several references.

Author response: *Thanks for this. The references have been formatted, corrected and volume numbers included in the references.*

### Reviewer’s report 2: Srikrishna Subramaniam, Institute of Microbial Technology/CSIR

In their manuscript entitled “Integrative modelling of TIR domain-containing adaptor molecule inducing interferon-β; (TRIF) provides insights into its auto-inhibited state” by Mahita and Sowdhamini, the authors present a plausible explanation for the auto-inhibition of TRIF via interaction with the TIR domain. The authors have used extensive docking followed by molecular dynamics of the N-terminal domain to the BB loop-B helix region of the TIR domain. Taken together the proposed mechanism seem convincing.

Minor issues

Please detail any minor comments for the authors attention (spelling, typographical errors, grammatical errors, stylistic suggestions etc.) so that, once addressed, the authors may remove them from the review.

I have some minor comments that I hope the authors will address.

1. The authors predict eight inter-domain residue pairs to be co-evolving. I suggest that they provide names of residues in addition to their position in Table 1. Do these residue pairs interact in both the predicted docked complexes as well as the MD simulated complexes?

Author response: *Thank you for the suggestions. We have now provided the names of the residues along with their positions in Table S1.*

These residue pairs do not interact in either the docked complexes or the MD simulated complexes. However, in some of these inter-domain residue pairs, one of the residues making up the co-evolving residue pair, is present at the interface. This could be due to the fact that we have fewer homologous sequences, which are not sufficient for predictions on the basis of residue pair correlations. We have specifically mentioned that the results of the co-evolution analysis needs to be interpreted with caution due to the lower number of sequences considered.

2. The docking study, as well as prior literature suggests an important structural role of the BB loop (residues 428-439) of the TIR domain for its interaction with the N-terminal domain.

a. The BB loop is highly flexible as observed in the 20 NMR models of TIR domain (PDBid: 2M1X). Have the authors accounted for the inherent flexibility of this loop during the docking experiments?

Author response: *This is a good point. We had performed docking using Cluspro, CAPSDOCK and HADDOCK. HADDOCK performs a short MD simulation on the binding site which includes the BB loop. Hence the flexibility of the BB loop has been taken into account during HADDOCK docking. Furthermore, following docking, molecular dynamics simulations of 1 microsecond have been performed to assess the stability of the complex. The flexibility of the BB loop is therefore considered during the MD simulations as well.*

b. The Methodology section says that a representative structure of the TIR (PDBid: 2M1X) was selected from the set of NMR structures. What criteria were considered to select the representative structure?

Author response: *According to the details provided in the PDB file (2M1X), the first NMR model is considered as the best representative conformer among all conformations in the NMR ensemble. Hence, we chose the first model for our analysis.*

c. The NMR structures of TIR domain (PDBid: 2M1X) is a Pro434His mutant structure. On what basis the phi-psi angles of the Proline have been taken in the modeled structure before docking? Kink forming residue like Proline is likely to induce a change in the backbone conformation. Would energy minimization of the protein be sufficient for attaining a local minimum conformation?

Author response: *The phi and psi angles of the corresponding proline in the BB loop of available TIR domain structures-TLR1,2,6 ,10 as well as MyD88 were first checked. Based on this information, the phi and psi values of the Pro 434 residue in the BB loop of TRIF were set. Energy minimization of the protein reduces steric clashes among the atoms and is sufficient to attain a local minimum if the minimization has converged. Further to energy minimisation, we carefully checked the local environment to ensure that there is no strain attributed to the fold.*

3. Structural modeling of the N-terminal domain-intermediate region-TIR domain does not add new insights. I suggest deleting this section.

Author response: Structural information is available for both N-terminal domain and TIR domain with high reliability since there are homologous protein domains with known structure. It is true that the intermediate region of TRIF is not relatively easy to predict structure. Further, this region is known to contain low-complexity and fairly unstructured regions further complicating the structure prediction. However, our fold prediction results are compelling and relates to the known fold of a viral protein with high confidence. Therefore, we went ahead and attempted modelling of this region as well. As is true with other viral proteins, like the coat proteins, this region – even in the template – has large structural embellishments. Therefore, the intermediate region might appear floppy. On the whole, we are also trying to obtain full-length-model of the TRIF protein, where two domains could be modelled with high reliability and the intermediate region is of relatively low accuracy (derived from fold prediction and contains few floppy regions). But, we trust it is still better than *not* having any structure of the full-length TRIF. The availability of full-length model of TRIF has also enabled us to examine the conformational flexibility through normal mode analyses. For these reasons, we would request that the intermediate region modelling be retained.

### Reviewer’s report 3: Peter Bond (nominated by Chandra Verma), Bioinformatics Institute, A*STAR, Singapore)

Whilst the authors use an original attempt to incorporate data from diverse sources, to come up with a reasonable working model for an important component of the innate immune system, the methodology used ranges from out-of-date to out-right disastrous. Thus, the validity of the results cannot be trusted, and the significance is minimal.

Reviewer recommendations to authors

Please make your report as constructive as possible, if necessary, recommending specific improvements so that the authors have the opportunity to overcome any serious deficiencies that you find. Please divide your comments into major and minor recommendations.

This article sets out to provide novel insights into the mechanism of autoinhibition of TRIF, by predicting the complex between its TIR domain and N terminal domain. This is potentially very worthwhile, and could be useful for the innate immunity community. However, I have some major concerns about the methodology that prevent this manuscript from being published, unfortunately…

1. The authors do a reasonable job of combining sources of data from evolutionary/mutational analysis of potentially interacting sites in each domain, docking, and comparison with available experimental data, leading them to propose that the N-terminal domain binds to the key BB loop of the TIR domain to prevent homodimerization, which seems reasonable. However, given the sparse data utilised to build this model, and the low result confidence, we need either new experiments to be performed to test the model, or EXTENSIVE simulations. Unfortunately, only one 50 ns and one 100 ns simulation has been used to test the stability of the model. This is far too limited sampling, by today’s standards. I would expect to see minimum of 1 microsecond of sampling, and at least 3-5 repeats of each system, to confirm the stability of the model and to check for disassembly or even unfolding, and to ensure the significance and reproducibility of the data.

Author response: *Thank you for your suggestions. We have now performed three replicates of MD simulations, each of 1 microsecond on the docked N-TIR complex and we find that in all the three replicates, the complex is stable and does not undergo disassembly or unfolding during the length of the entire simulations. The hydrogen bonds present at the interface are also conserved between replicates.*

2. Even for the simulations that have been performed, there is extremely limited quantitative analysis presenting with regards the stability. I would expect to see basic conformational/structural analysis, such as time-dependent RMSDs, radii of gyration, solvent-accessible and buried areas, etc….. do we simply have to trust the conclusions without seeing any evidence??

Author response: *Thanks for this suggestion. The basic analyses (RMSD,RMSF, Radius of Gyration) performed on the MD simulations has now been included in the Supplementary Materials section of the revised version.*

3. If I understand the manuscript correctly, the second half is concerned with modelling the linker between the N-termianl domain and TIR domain. This spans residues 154-391… i.e. over 200 residues! Because no protein of known structure with similar sequence is available, fold-recognition approaches were used to identify a possible protein to serve as a template for homology modelling. Frankly, this is pure fantasy. Even for a template with high sequence identity (e.g. >30%), it can be tough to correctly predict its structure. To do so for a template without identity, and an intrinsically disordered region at that, is utterly impossible. All the subsequent results based on this model are likely to be junk, and should be discarded.

Author response: Structural information is available for both N-terminal domain and TIR domain with high reliablility since there are homologous protein domains with known structure. It is true that the intermediate region of TRIF is not relatively easy to predict structure. Further, this region is known to contain low-complexity and fairly unstructured regions, thereby complicating the structure prediction. However, our fold prediction results are compelling and relates to the known fold of a viral protein with high confidence. Therefore, we went ahead and attempted modelling of this region as well. As is true with other viral proteins, like the coat proteins, this region – even in the template – has large structural embellishments. The intermediate region might thus appear floppy. On the whole, we are also trying to obtain this full-length-model of the TRIF protein, where two domains could be modelled with high reliability and the intermediate region is of relatively low accuracy (derived from fold prediction and contains few floppy regions). But, we trust it is still better than *not* having any structure of the full-length TRIF. The availability of full-length model of TRIF has also enabled us to examine the conformational flexibility through normal mode analyses. It is nowadays acceptable to present models of a protein, where the reliability of model is not uniformly strong – it is indeed the power of integrated modelling approaches like these. If we examine the solution of electron microscopy data of large assemblies, we frequently encounter this situation.

4. Apparently geometrical/stereochemical/structural/energetic checks were performed for the models built in this manuscript. No such data was actually presented… again, should the reader simply trust the conclusions without seeing any evidence?

Author response: *The basic MD validation checks and analyses (such as RMSD, RMSF, Radius of Gyration) had indeed been performed on our MD simulations and now for the triplicates of long-length simulations. These are now being included in the Supplementary Materials section of the revised version. Thanks for this comment.*

Minor issues

Please detail any minor comments for the authors attention (spelling, typographical errors, grammatical errors, stylistic suggestions etc.) so that, once addressed, the authors may remove them from the review.

Model coordinates should be made available.

Author response: *Model coordinates of the N-TIR complex have been provided.*

“Molecular dynamic simulations” should be “molecular dynamics simulations”. There are various other grammatical errors throughout the manuscript.

Author response: *The spelling error and other grammatical errors have been rectified.*

I would have liked to see some critical analysis of the results of the mutual information analysis. How much confidence can we really have in the results? This is important since all the subsequent results and models are reliant on this data.

Author response: *The subsequent results and models are not reliant on the mutual information analysis due to paucity of number of sequence homologues. While the co-evolution analysis is based entirely on sequence information alone, the models generated through docking are based on known experimental data, conservation of residues and the information obtained from the available structures of the N-terminal domain and the TIR domain of TRIF. The results of the co-evolution analysis complements the results of docking.*

Although published, at least short descriptions of the underlying methodology for the various “in-house algorithms” used in this paper should have been provided, e.g. for Dockscore, PPCheck, etc.

Author response: *Thanks for this comment. Additional details of the in-house algorithms used in this work are now provided in the revised version.*

### Reviewers’ response to authors after revision

#### Peter Bond

With the new simulation data, I am mostly satisfied with the authors’ revised manuscript, and I think it is a nice body of work, with one continuing exception. I still cannot agree with the section on modelling the linker between N-terminal and TIR domain. Ab initio modelling of a 200 residue segment based on fold-recognition alone, in the absence of similar template structures, is, as I stated previously, pure fantasy, in my opinion. This is compounded by the likely intrinsically disordered nature of the segment. I note that I am not alone in having this opinion, as reviewer 2 states, “Structural modeling of the N-terminal domain-intermediate region-TIR domain does not add new insights. I suggest deleting this section.” The author response was that “we trust it is still better than *not* having any structure of the full-length TRIF.” I cannot agree with this philosophy - in fact, this is precisely the opposite of my own judgement. Publishing something that is likely to be wrong is worse than publishing nothing at all.

Our response: In order to honour the referee’s continued concern on this point, we are happy to remove this part – middle domain modelling and normal mode analysis of the full-length protein out of our manuscript.

## Additional files

Additional file 1: **Appendix S1.** List of organisms used for co-evolution analysis (DOCX 16 kb)

Additional file 2: Figure S1. The concatenated multiple sequence alignment of the N-terminal domain and TIR domain sequences from different organisms used for predicting co-evolving pair of residues. The region of the MSA corresponding to the N-terminal domain sequences is highlighted in purple while the region corresponding to the TIR domain is highlighted in green. Different colours for different co-evolving pair of residues with one colour for each pair have also been marked on the alignment. (PDF 4 MB)

Additional file 3: Table S1. Co-evolving pair of residues predicted by CMAT. The N-terminal domain corresponds to residues 1 to 153, while residues 154–349 make up the TIR domain in the concatenated multiple sequence alignment. (DOCX 16 kb)

Additional file 4: **Figure S2a.** TIR domain of TRIF showing the position of the BB loop, B helix and the RK site. **Figure S2b.** Electrostatic surface potential representation of the TRIF TIR domain. (ZIP 17210 kb)

Additional file 5: Figure S3. Multiple sequence alignment of the N-terminal domain of TRIF from mammals, birds and fishes. Conserved polar residues, clustering around Regions 1 and 2 are marked in light brown and cyan colours respectively. (TIF 23000 kb)

Additional file 6: Table S2. Summary of interface residues identified in the different models generated by semi-guided docking using the Cluspro docking program. (DOCX 13 kb)

Additional file 7: Figure S4. Our model of the N-terminal protease-resistant domain docked onto the TIR domain. The N-terminal domain is coloured in purple and the TIR domain in green. (TIF 3811 kb)

Additional file 9: **Figure S5a.** A plot showing the variation in backbone RMSD along the trajectory, for each of the three replicates. **Figure S5b.** Plot of the radius of gyration for each of the three replicates. **Figure S5c.** The root mean square fluctuations of backbone atoms, averaged over the last 200 ns (800 ns-1000 ns) of the simulations, for each replicate. (ZIP 13857 kb)

Additional file 10: **Figure S6a.** Profile of hydrogen bonds between Asp 21 on the N-terminal domain and Gln 443 on the TIR domain of TRIF along the MD trajectory. **Figure S6b.** Hydrogen bonds between Asp 21 and Leu 442 along the trajectory. **Figure S6c.** Hydrogen bonds between Lys 22 and Gln 471 over the MD trajectory. **Figure S6d.** Hydrogen bonds between Gln 20 and BB loop residue Glu 429. (ZIP 4528 kb)

Additional file 11: **Figure S7a.** The hydrogen bond energy at the interface of the N-TIR complex calculated by PPCheck from different snapshots extracted every 1 ns from the trajectory, for all three replicates. **Figure S7b.** Variation in PPCheck-derived electrostatic energy at the interface during the course of the three MD simulations. **Figure S7c**:. van der Waals energies at the interface, calculated using PPCheck. **Figure S7d.** Total stabilizing energy at the interface. **Figure S7e.** Relative variation in the number of residues present at the interface. **Figure S7f.** Normalized energy per residue, as calculated using the PPCheck algorithm, at the interface. (ZIP 3290 kb)

Additional file 12: Figure S8. Predicted co-evolving pairs of residues mapped onto the N-TIR docked complex. The colouring scheme follows the same scheme as in Additional file 3: Figure S1. (TIF 6339 kb)

Additional file 13: Model coordinates of the N-TIR complex are provided as a Supplementary Material. (PDB 236 kb)

Additional file 14: Figure S9. Our model of the mechanism of TRIF autoinhibition. (a). Upon TLR3 activation, the 522R/523K (RK) site of the TIR domain of TRIF binds to the dimerised TIR domains of the TLR3 dimer. (b). This induces a conformational change mediated by the long-range interactions between the BB loop and RK site of TRIF, exposing the BB loop. (c). This facilitates oligomerisation of TRIF leading to downstream signalling. (TIF 272014 kb)

## Abbreviations

CMAT: Correlated Mutation Analysis Tool
DAMPS: Danger-associated molecular patterns
IFIT: Interferon-induced proteins with tetracotripeptide repeats
IRF: Interferon regulatory factor
MAL/TIRAP: MyD88 adaptor-like/TIR domain-containing adaptor protein
MAVS: Mitochondrial antiviral signaling protein
MI: Mutual information
MyD88: Myeloid differentiation primary reponse gene 88
NF-kB: Nuclear factor kappa-light chain enhancer of activated B cells
PAMPS: Pathogen-associated molecular patterns
PSN: Protein Structure Network
RHIM: Receptor homotypic interaction motif
SARM: Sterile-α and HEAT-Armadillo motifs containing protein
TBK1: TANK-binding kinase 1
TIR: Toll/Interleukin-1 receptor homology
TLR3: Toll-like receptor 3
TLR4: Toll-like receptor 4
TLRs: Toll-like receptors
TPRs: Tetracotripeptide repeats
TRAF2: Tumour necrosis factor (TNF) receptor associated factor 2
TRAF6: Tumour necrosis factor (TNF) receptor associated factor 6
TRAM/TICAM-2: TRIF-related adaptor molecule/TIR domain-containing adaptor molecule 2
TRIF/TICAM-1: TIR domain-containing adaptor molecule-inducing interferon-β/TIR domain-containing molecule 1
