# Supplementary material for: Integrative modelling of TIR domain-containing adaptor molecule inducing interferon-β (TRIF) provides insights into its autoinhibited state
Source: Biol Direct. 2017 Apr 20;12:9. doi: 10.1186/s13062-017-0179-0 (PMC5397763; doi:10.1186/s13062-017-0179-0)
Supplement: Supplementary file 2 — Appendix S1. List of organisms used for co-evolution analysis (DOCX 16 kb) [file 13062_2017_179_MOESM1_ESM.docx]

1. *Homo sapiens*
2. *Gorilla gorilla*
3. *Pan paniscus*
4. *Pan troglodytes*
5. *Pongo pygmaeus*
6. *Pongo abelii*
7. *Nomascus leucogenys*
8. *Cercocebus atys*
9. *Macaca fascicularis*
10. *Macaca mulatta*
11. *Mandrillus leucophaeus*
12. *Chlorocebus sabaeus*
13. *Rhinopithecus roxellana*
14. *Papio anubis*
15. *Colobus angolensis palliatus*
16. *Aotus nancymaae*
17. *Saimiri boliviensis boliviensi*
18. *Callithrix jacchus*
19. *Propithecus coquereli*
20. *Galeopterus variegatus*
21. *Otolemur garnettii*
22. *Microcebus murinus*
23. *Ceratotherium simum simum*
24. *Vicugna pacos*
25. *Bubalus bubalis*
26. *Bos taurus*
27. *Bos mutus*
28. *Capra hircus*
29. *Sus scrofa*
30. *Equus caballus*
31. *Canis lupus familiaris isoform1*
32. *Ovis aries*
33. *Ovis aries musimon*
34. *Mustela putorius furo*
35. *Bison bison bison*
36. *Leptonychotes weddellii*
37. *Camelus ferus*
38. *Camelus dromedarius*
39. *Camelus bactrianus*
40. *Pteropus alecto*
41. *Pteropus vampyrus*
42. *Pantholops hodgsonii*
43. *Physeter catodon*
44. *Orcinus orca*
45. *Balaenoptera acutorostrata sca*
46. *Eptesicus fuscus*
47. *Myotis lucifugus*
48. *Myotis davidii*
49. *Ictidomys tridecemlineatus*
50. *Lipotes vexillifer*
51. *Tursiops truncatus*
52. *Trichechus manatus latirostris*
53. *Odobenus rosmarus divergens*
54. *Orycteropus afer afer*
55. *Ursus maritimus*
56. *Ailuropoda melanoleuca*
57. *Felix catus*
58. *Panthera tigris altaica*
59. *Loxodonta africana*
60. *Jaculus jaculus*
61. *Octodon degus*
62. *Dipodomys ordii*
63. *Cricetulus griseus*
64. *Mesocricetus auratus*
65. *Fukomys damarensis*
66. *Nannospalax galili*
67. *Heterocephalus glaber*
68. *Chinchilla lanigera*
69. *Microtus ochrogaster*
70. *Chrysochloris asiatica*
71. *Mus musculus*
72. *Peromyscus maniculatus bairdii*
73. *Elephantulus edwardii*
74. *Cavia porcellus*
75. *Echinops telfairi*
76. *Rattus norvegicus*
77. *Ochotona princeps*
78. *Condylura cristata*
79. *Sorex araneus*
80. *Tupaia chinensis*
81. *Erinaceus europaeus*
82. *Monodelphis domestica*
83. *Sarcophilus harrisii*
84. *Dasypus novemcinctus*
85. *Ornithorhynchus anatinus*
86. *Taeniopygia guttata*
87. *Latimeria chalumnae*
88. *Gallus gallus*
89. *Meleagris gallopavo*
90. *Ficedula albicollis*
91. *Anas platyrhynchos*
92. *Anolis carolinensis*
93. *Ophiophagus hannah*
94. *Takifugu rubripes*
95. *Poecilia formosa*
96. *Danio rerio*
97. *Ctenopharyngodon idella*
