# Supplementary material for: Integrative modelling of TIR domain-containing adaptor molecule inducing interferon-β (TRIF) provides insights into its autoinhibited state
Source: Biol Direct. 2017 Apr 20;12:9. doi: 10.1186/s13062-017-0179-0 (PMC5397763; doi:10.1186/s13062-017-0179-0)
Supplement: Supplementary file 4 — Co-evolving pair of residues predicted by CMAT. The N-terminal domain corresponds to residues 1 to 153, while residues 154–349 make up the TIR domain in the concatenated multiple sequence alignment. (DOCX 16 kb) [file 13062_2017_179_MOESM3_ESM.docx]

**Table S1.**

Co-evolving pair of residues predicted by CMAT. The N-terminal domain corresponds to residues 1 to 153, while residues 154-349 make up the TIR domain in the concatenated multiple sequence alignment.

| S. No | i | AA at i | j | AA  at j | Neff | MI | NMI | MIp | Zp | MIc | Zc |
| --- | --- | --- | --- | --- | --- | --- | --- | --- | --- | --- | --- |
| 1 | 138 | E | 302 | I | 3.15 | 0.555 | 0.08 | 0.226 | 4.673 | 0.189 | 4.373 |
| 2 | 212 | S | 300 | K | 3.3 | 0.298 | 0.052 | 0.184 | 3.815 | 0.185 | 4.296 |
| 3 | 226 | R | 273 | L | 3.34 | 0.473 | 0.078 | 0.204 | 4.211 | 0.184 | 4.275 |
| 4 | 43 | L | 169 | E | 3.05 | 0.393 | 0.073 | 0.195 | 4.036 | 0.183 | 4.254 |
| 5 | 12 | F | 117 | A | 3.2 | 0.288 | 0.052 | 0.181 | 3.744 | 0.179 | 4.16 |
| 6 | 54 | E | 222 | N | 3.22 | 0.429 | 0.072 | 0.192 | 3.981 | 0.178 | 4.148 |
| 7 | 231 | Q | 290 | T | 3.32 | 0.37 | 0.065 | 0.186 | 3.857 | 0.178 | 4.146 |
| 8 | 30 | L | 70 | A | 3.1 | 0.317 | 0.06 | 0.183 | 3.789 | 0.178 | 4.135 |
| 9 | 213 | A | 245 | K | 3.34 | 0.296 | 0.049 | 0.172 | 3.556 | 0.176 | 4.094 |
| 10 | 102 | L | 290 | T | 3.09 | 0.343 | 0.064 | 0.183 | 3.793 | 0.175 | 4.079 |
| 11 | 49 | L | 58 | R | 3.22 | 0.5 | 0.089 | 0.191 | 3.948 | 0.171 | 3.983 |
| 12 | 51 | L | 57 | A | 3.22 | 0.222 | 0.044 | 0.172 | 3.549 | 0.17 | 3.97 |
| 13 | 98 | R | 118 | Y | 3.1 | 0.334 | 0.057 | 0.175 | 3.616 | 0.17 | 3.959 |
| 14 | 336 | Y | 339 | S | 3.19 | 0.856 | 0.132 | 0.245 | 5.075 | 0.166 | 3.883 |
| 15 | 34 | R | 54 | E | 2.69 | 0.492 | 0.074 | 0.195 | 4.029 | 0.166 | 3.875 |
| 16 | 319 | L | 322 | E | 3.13 | 0.581 | 0.095 | 0.204 | 4.215 | 0.164 | 3.83 |
| 17 | 156 | K | 216 | I | 3.17 | 0.448 | 0.076 | 0.182 | 3.757 | 0.163 | 3.812 |
| 18 | 337 | L | 341 | L | 3.19 | 0.771 | 0.112 | 0.235 | 4.856 | 0.161 | 3.778 |
| 19 | 52 | G | 184 | V | 3.18 | 0.448 | 0.083 | 0.179 | 3.692 | 0.161 | 3.776 |
| 20 | 136 | L | 156 | K | 3.04 | 0.573 | 0.094 | 0.192 | 3.964 | 0.155 | 3.643 |
| 21 | 28 | H | 55 | T | 3.22 | 0.578 | 0.093 | 0.185 | 3.835 | 0.15 | 3.545 |
| 22 | 19 | P | 267 | P | 3.1 | 0.439 | 0.074 | 0.174 | 3.594 | 0.15 | 3.534 |
| 23 | 149 | I | 166 | A | 2.95 | 0.612 | 0.088 | 0.206 | 4.261 | 0.149 | 3.527 |
| 24 | 323 | R | 332 | A | 3.07 | 0.735 | 0.116 | 0.208 | 4.312 | 0.149 | 3.525 |

**i and j**: positions(i < j)

**AA**: amino acid

**Neff:** effective number of sequences that aligned at the positions

**MI**: mutual information

**NMI**: normalized mutual information

**MIp and Zp**: average-product correction score and its Z-score

**MIc and Zc:** coevolution-pattern-similarity correction score and its Z-score
