# Supplementary figures and images for: Integrative modelling of TIR domain-containing adaptor molecule inducing interferon-β (TRIF) provides insights into its autoinhibited state
Source: Biol Direct. 2017 Apr 20;12:9. doi: 10.1186/s13062-017-0179-0 (PMC5397763; doi:10.1186/s13062-017-0179-0)

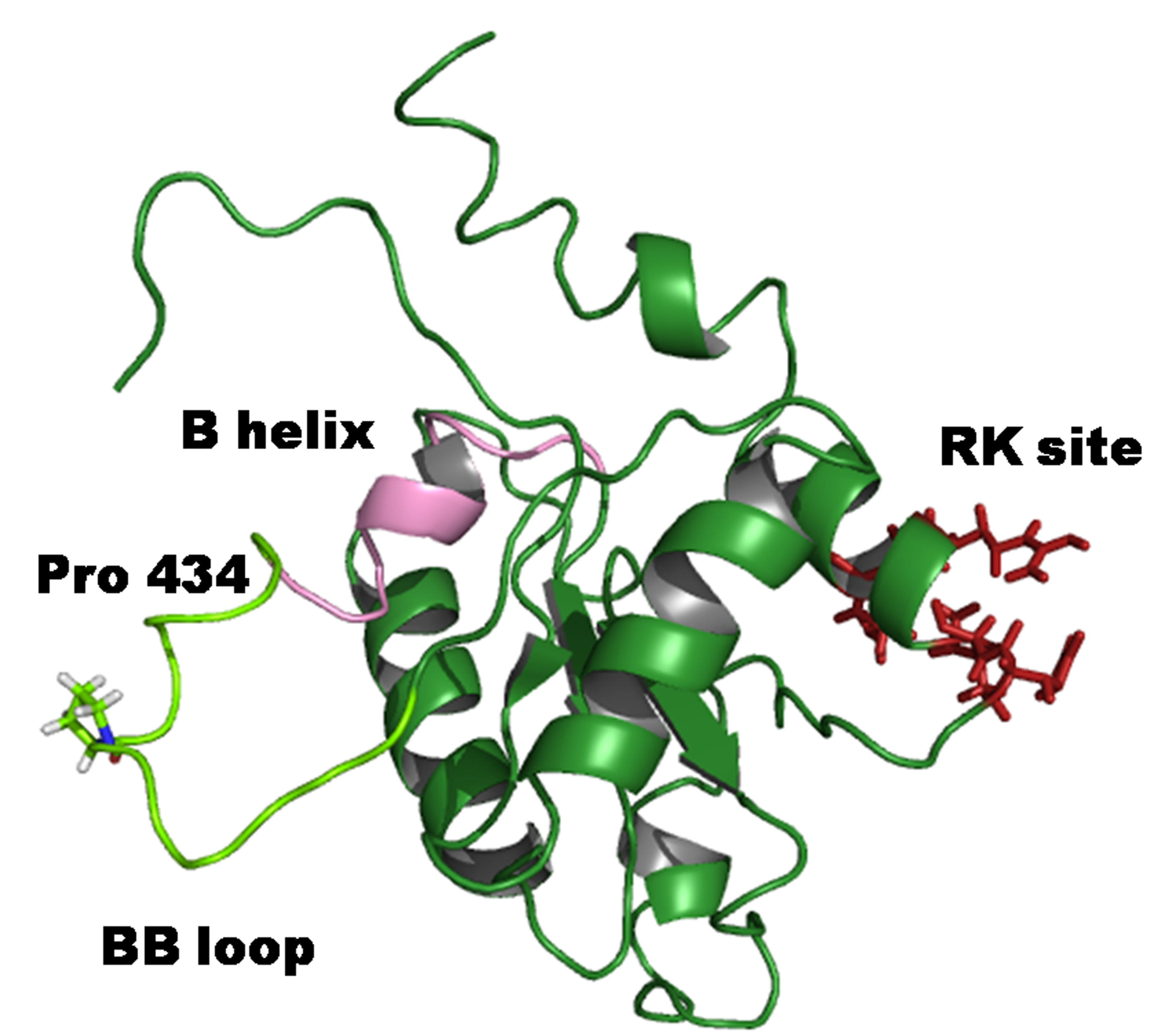

Supplement: Supplementary file 5 — Figure S2a. TIR domain of TRIF showing the position of the BB loop, B helix and the RK site. Figure S2b. Electrostatic surface potential representation of the TRIF TIR domain. (ZIP 17210 kb) [file 13062_2017_179_MOESM4_ESM.zip › 4/Figure_S2aR2.tif]

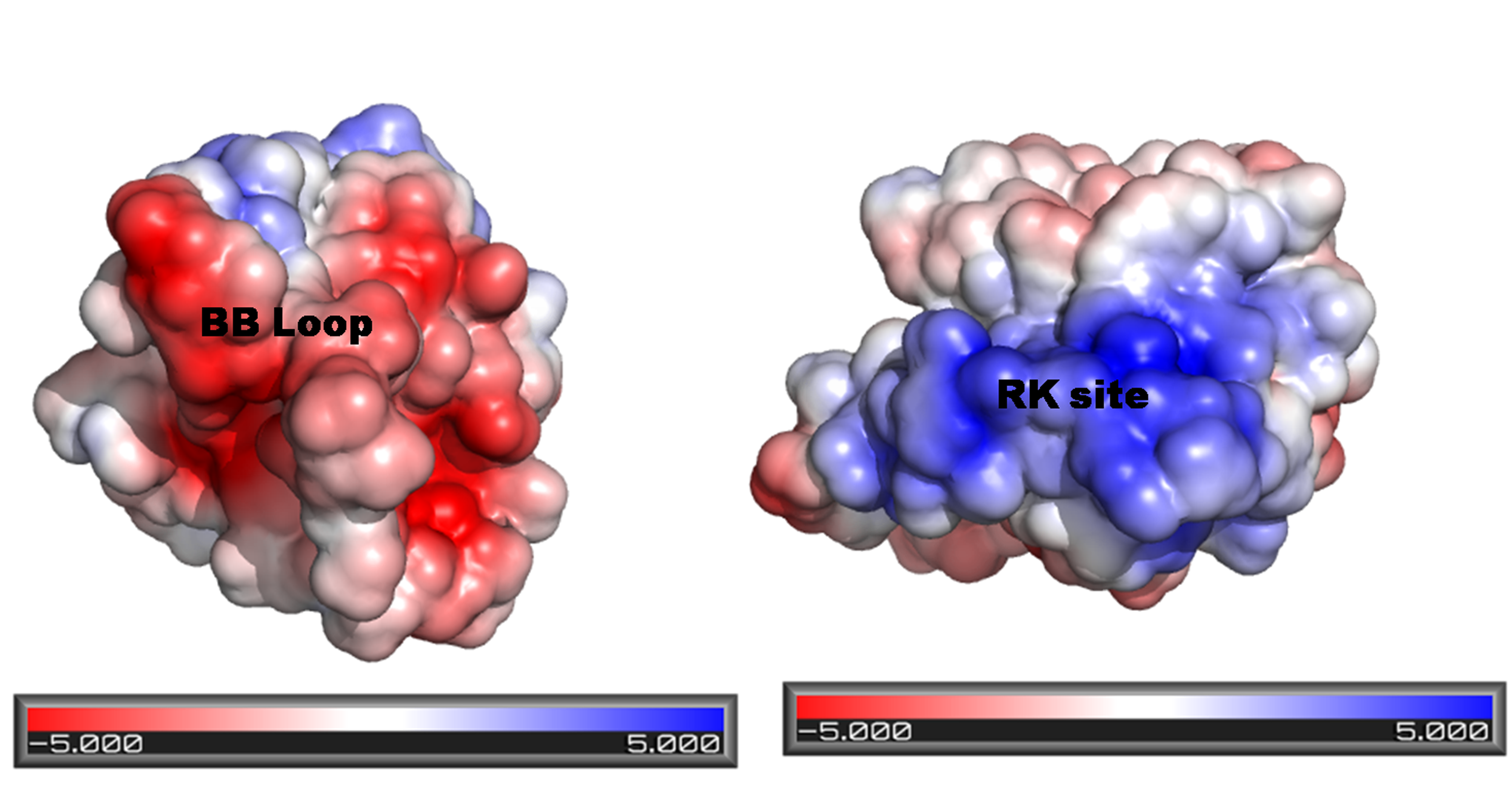

Supplement: Supplementary file 5 — Figure S2a. TIR domain of TRIF showing the position of the BB loop, B helix and the RK site. Figure S2b. Electrostatic surface potential representation of the TRIF TIR domain. (ZIP 17210 kb) [file 13062_2017_179_MOESM4_ESM.zip › 4/Figure_S2bR2.tif]

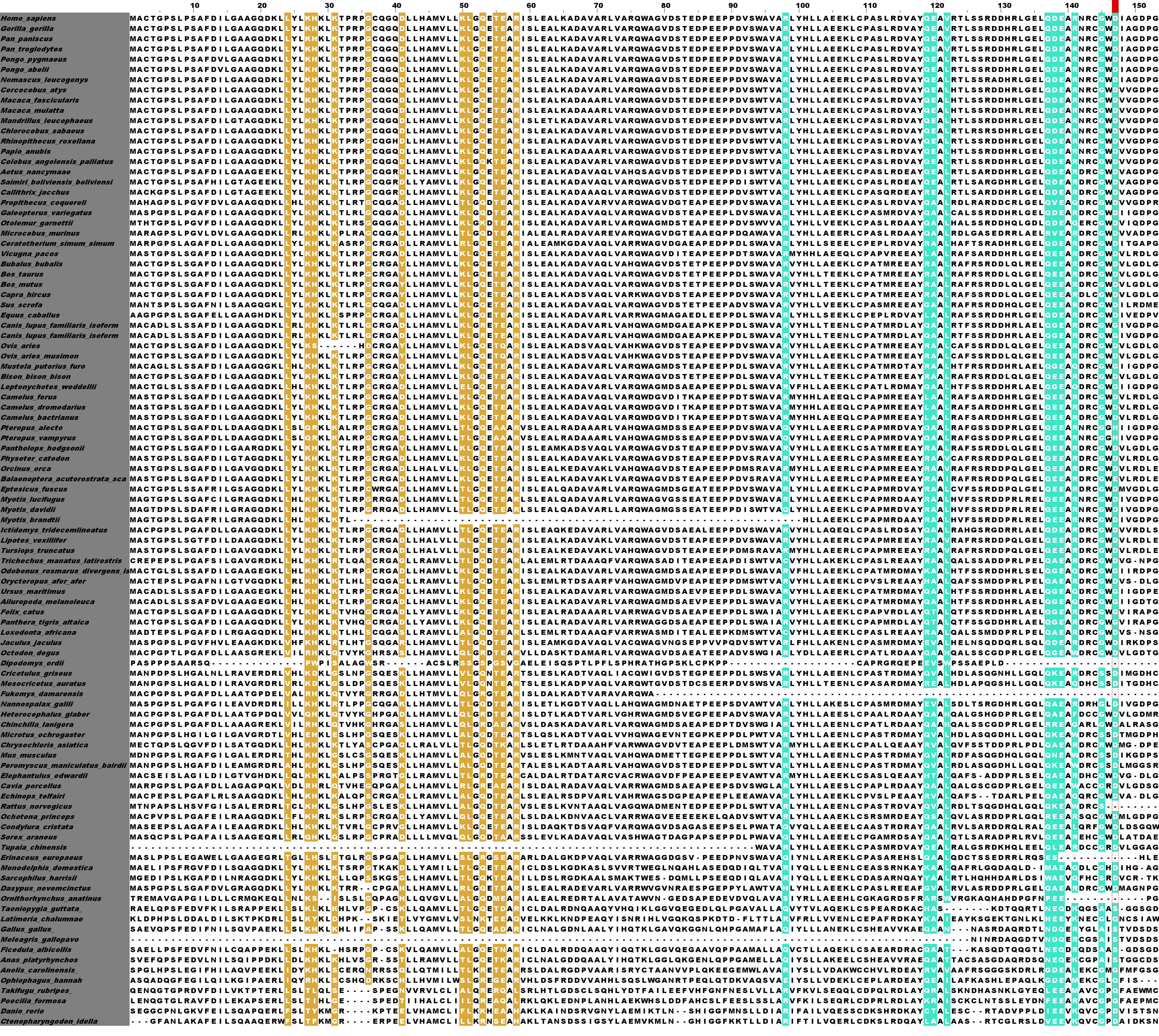

Supplement: Supplementary file 6 — Multiple sequence alignment of the N-terminal domain of TRIF from mammals, birds and fishes. Conserved polar residues, clustering around Regions 1 and 2 are marked in light brown and cyan colours respectively. (TIF 23000 kb) [file 13062_2017_179_MOESM5_ESM.tif]

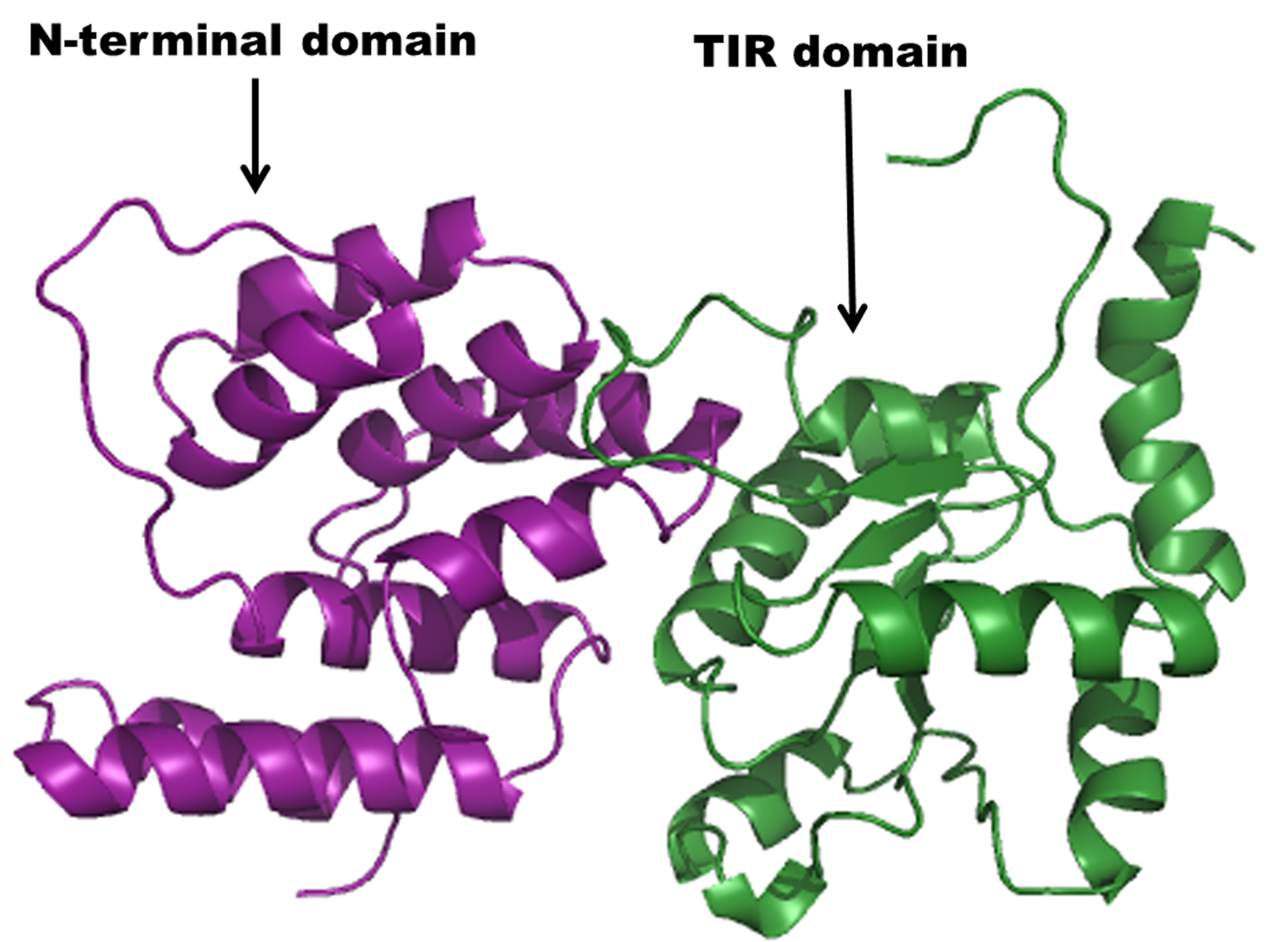

Supplement: Supplementary file 8 — Our model of the N-terminal protease-resistant domain docked onto the TIR domain. The N-terminal domain is coloured in purple and the TIR domain in green. (TIF 3811 kb) [file 13062_2017_179_MOESM7_ESM.tif]

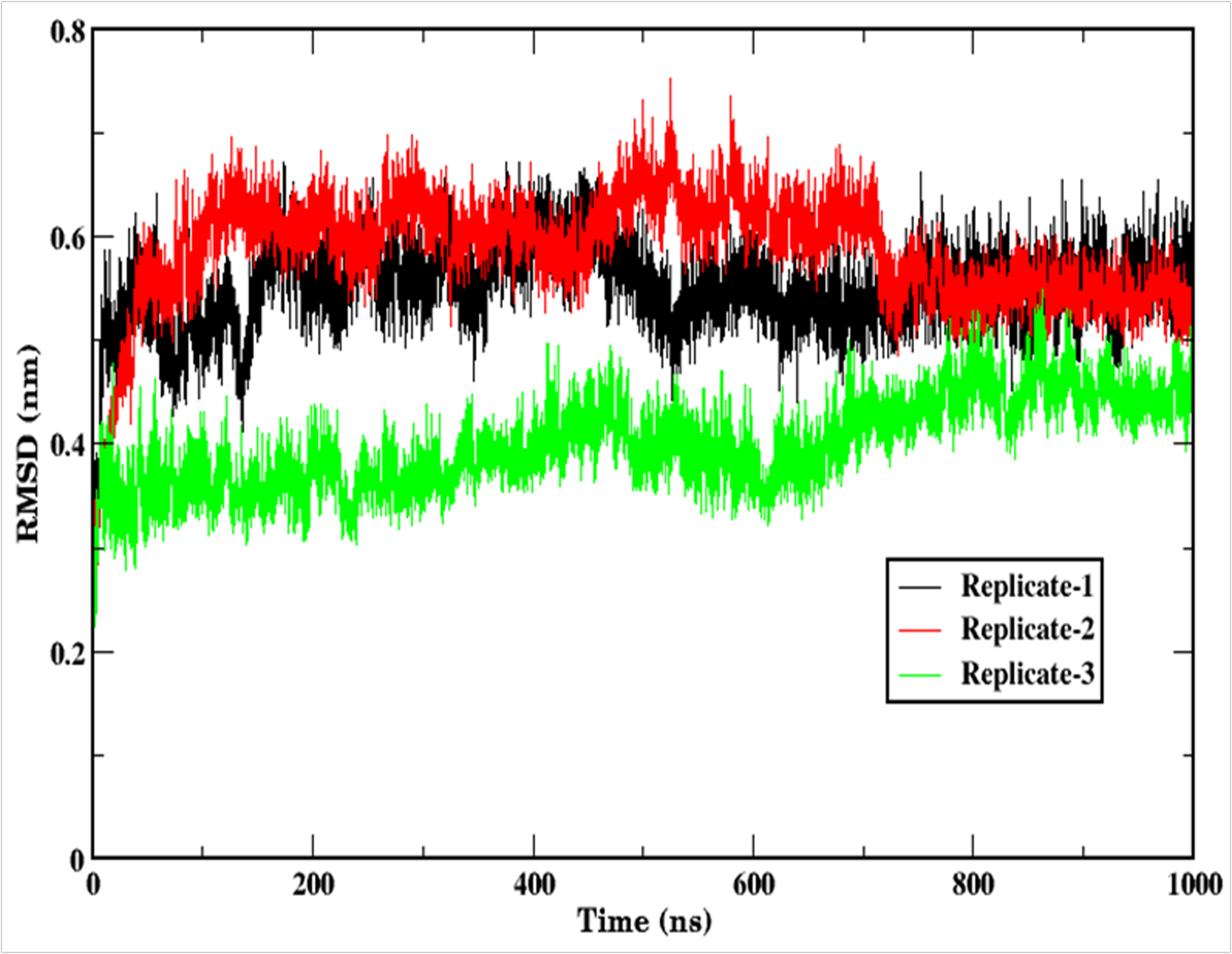

Supplement: Supplementary file 9 — Figure S5a. A plot showing the variation in backbone RMSD along the trajectory, for each of the three replicates. Figure S5b. Plot of the radius of gyration for each of the three replicates. Figure S5c. The root mean square fluctuations of backbone atoms, averaged over the last 200 ns (800 ns-1000 ns) of the simulations, for each replicate. (ZIP 13857 kb) [file 13062_2017_179_MOESM9_ESM.zip › 9/Figure_S5aR2_600.tif]

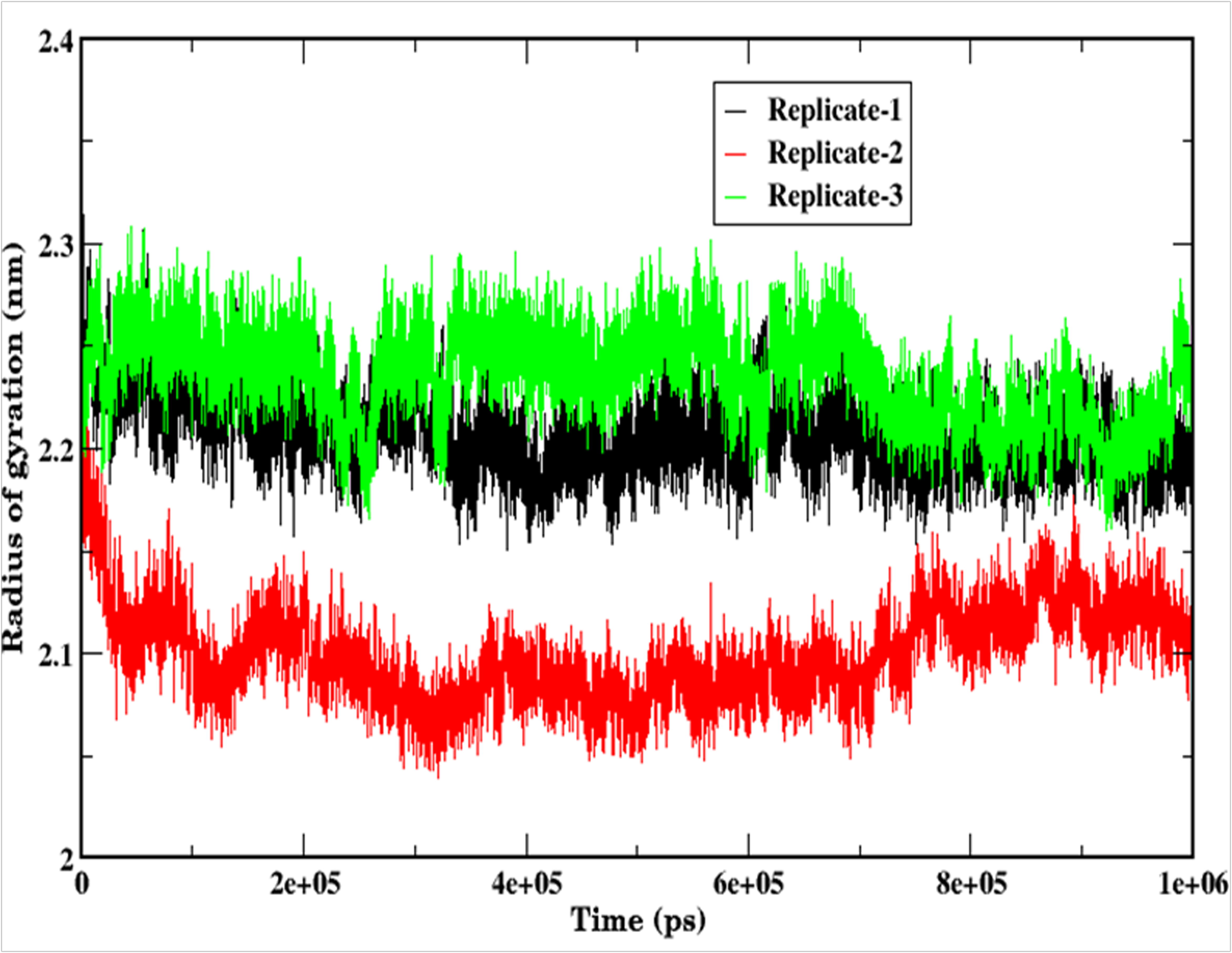

Supplement: Supplementary file 9 — Figure S5a. A plot showing the variation in backbone RMSD along the trajectory, for each of the three replicates. Figure S5b. Plot of the radius of gyration for each of the three replicates. Figure S5c. The root mean square fluctuations of backbone atoms, averaged over the last 200 ns (800 ns-1000 ns) of the simulations, for each replicate. (ZIP 13857 kb) [file 13062_2017_179_MOESM9_ESM.zip › 9/Figure_S5bR2_600.tif]

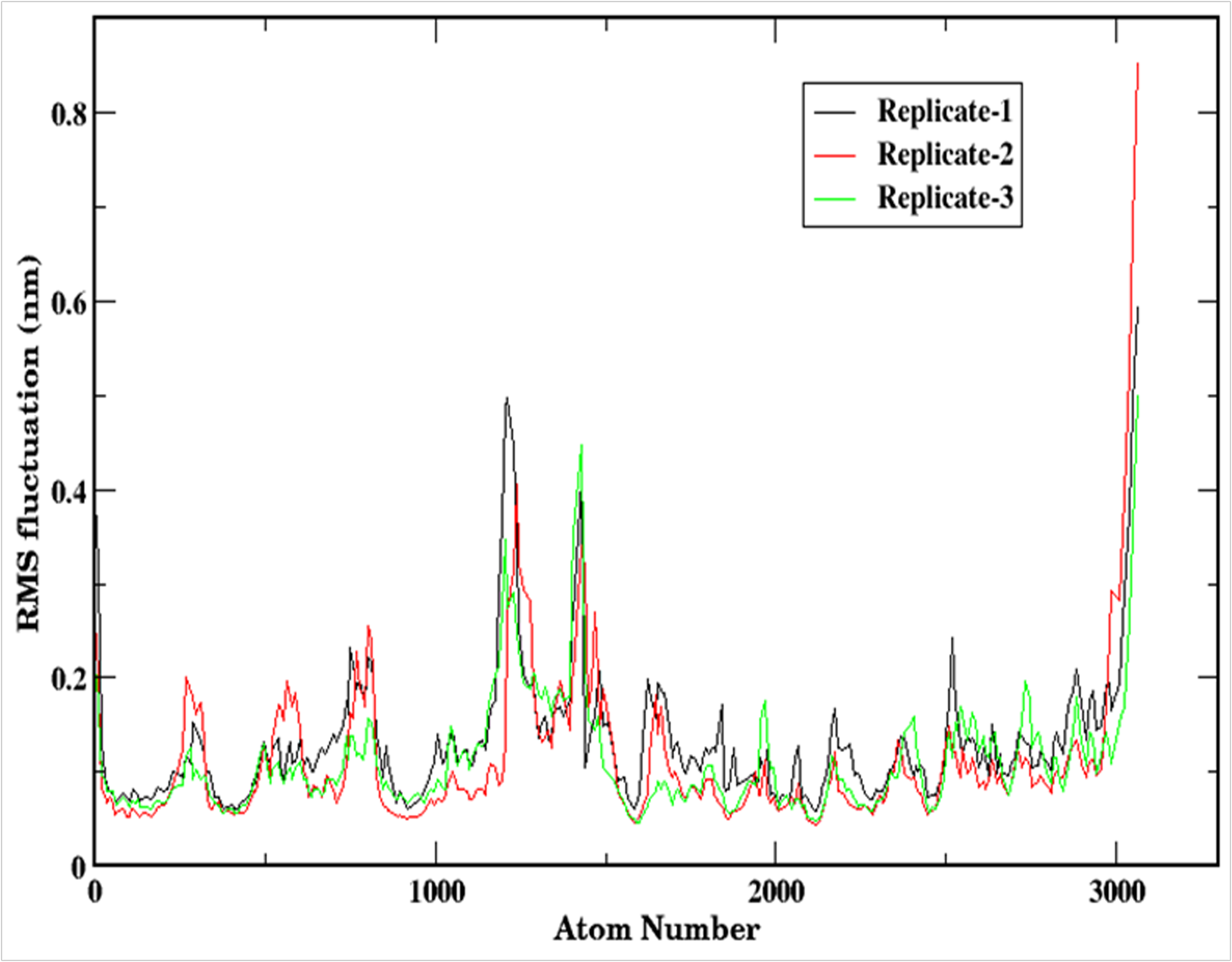

Supplement: Supplementary file 9 — Figure S5a. A plot showing the variation in backbone RMSD along the trajectory, for each of the three replicates. Figure S5b. Plot of the radius of gyration for each of the three replicates. Figure S5c. The root mean square fluctuations of backbone atoms, averaged over the last 200 ns (800 ns-1000 ns) of the simulations, for each replicate. (ZIP 13857 kb) [file 13062_2017_179_MOESM9_ESM.zip › 9/Figure_S5cR2_600.tif]

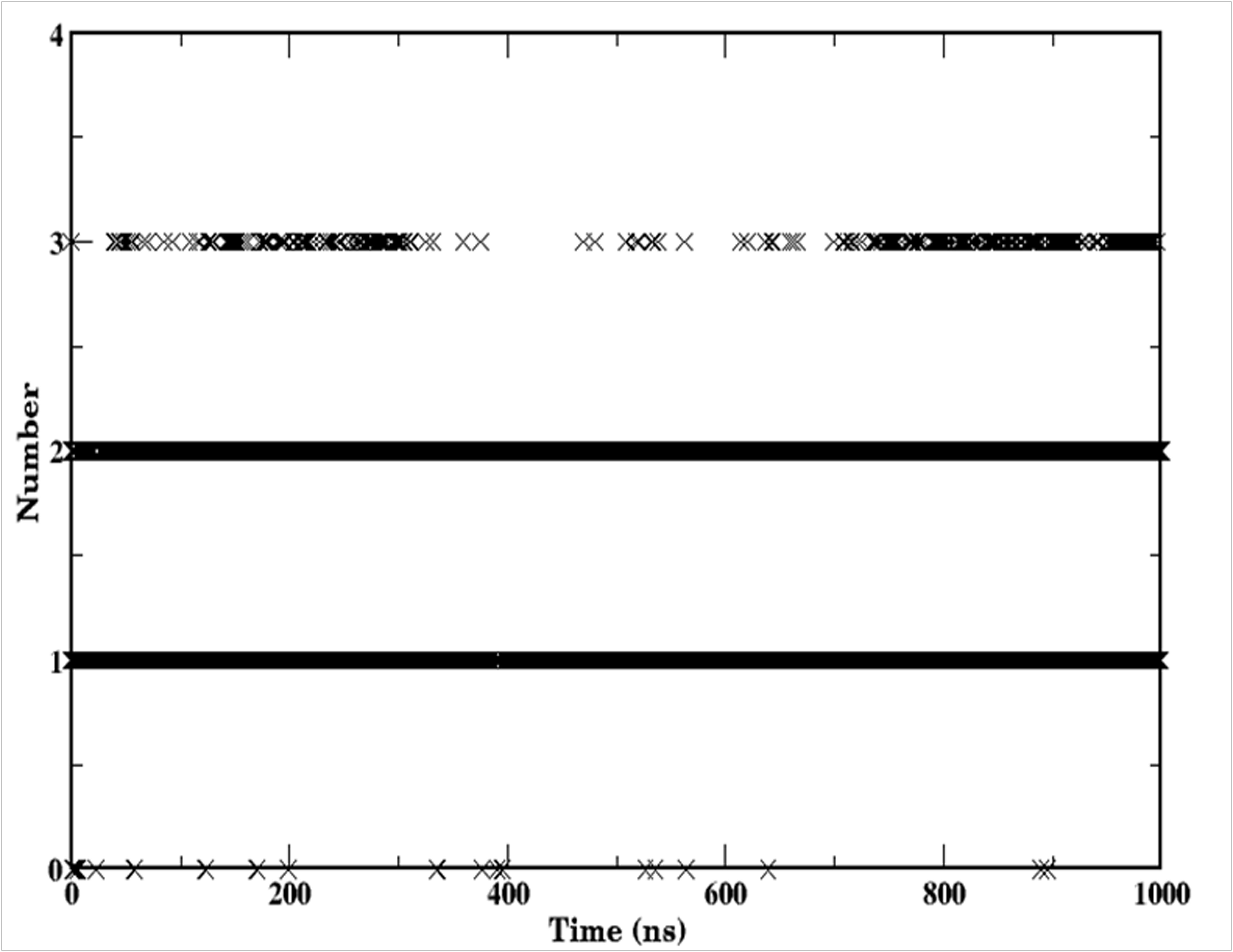

Supplement: Supplementary file 10 — Figure S6a. Profile of hydrogen bonds between Asp 21 on the N-terminal domain and Gln 443 on the TIR domain of TRIF along the MD trajectory. Figure S6b. Hydrogen bonds between Asp 21 and Leu 442 along the trajectory. Figure S6c. Hydrogen bonds between Lys 22 and Gln 471 over the MD trajectory. Figure S6d. Hydrogen bonds between Gln 20 and BB loop residue Glu 429. (ZIP 4528 kb) [file 13062_2017_179_MOESM10_ESM.zip › 10/Figure_S6aR2_600.tif]

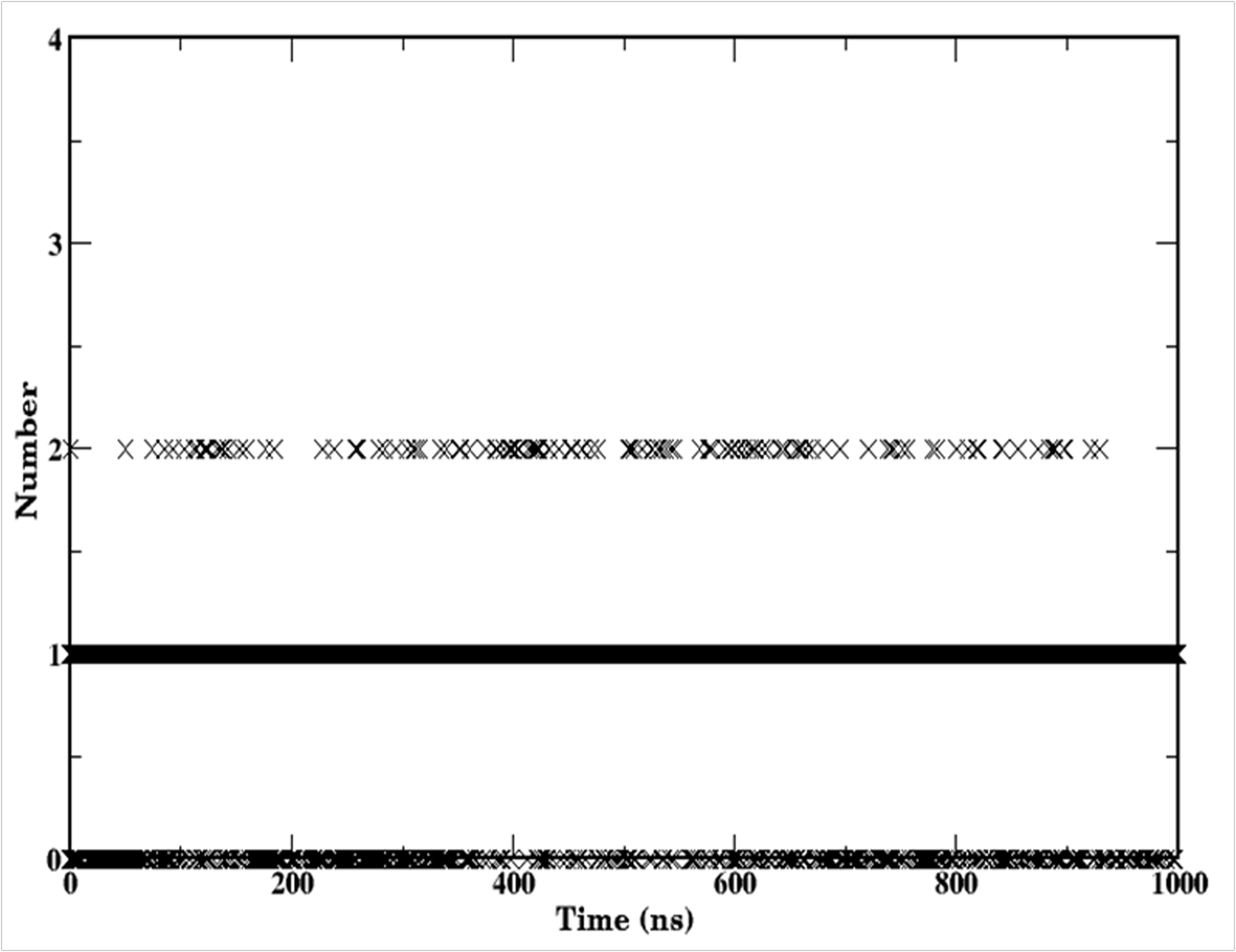

Supplement: Supplementary file 10 — Figure S6a. Profile of hydrogen bonds between Asp 21 on the N-terminal domain and Gln 443 on the TIR domain of TRIF along the MD trajectory. Figure S6b. Hydrogen bonds between Asp 21 and Leu 442 along the trajectory. Figure S6c. Hydrogen bonds between Lys 22 and Gln 471 over the MD trajectory. Figure S6d. Hydrogen bonds between Gln 20 and BB loop residue Glu 429. (ZIP 4528 kb) [file 13062_2017_179_MOESM10_ESM.zip › 10/Figure_S6bR2_600.tif]

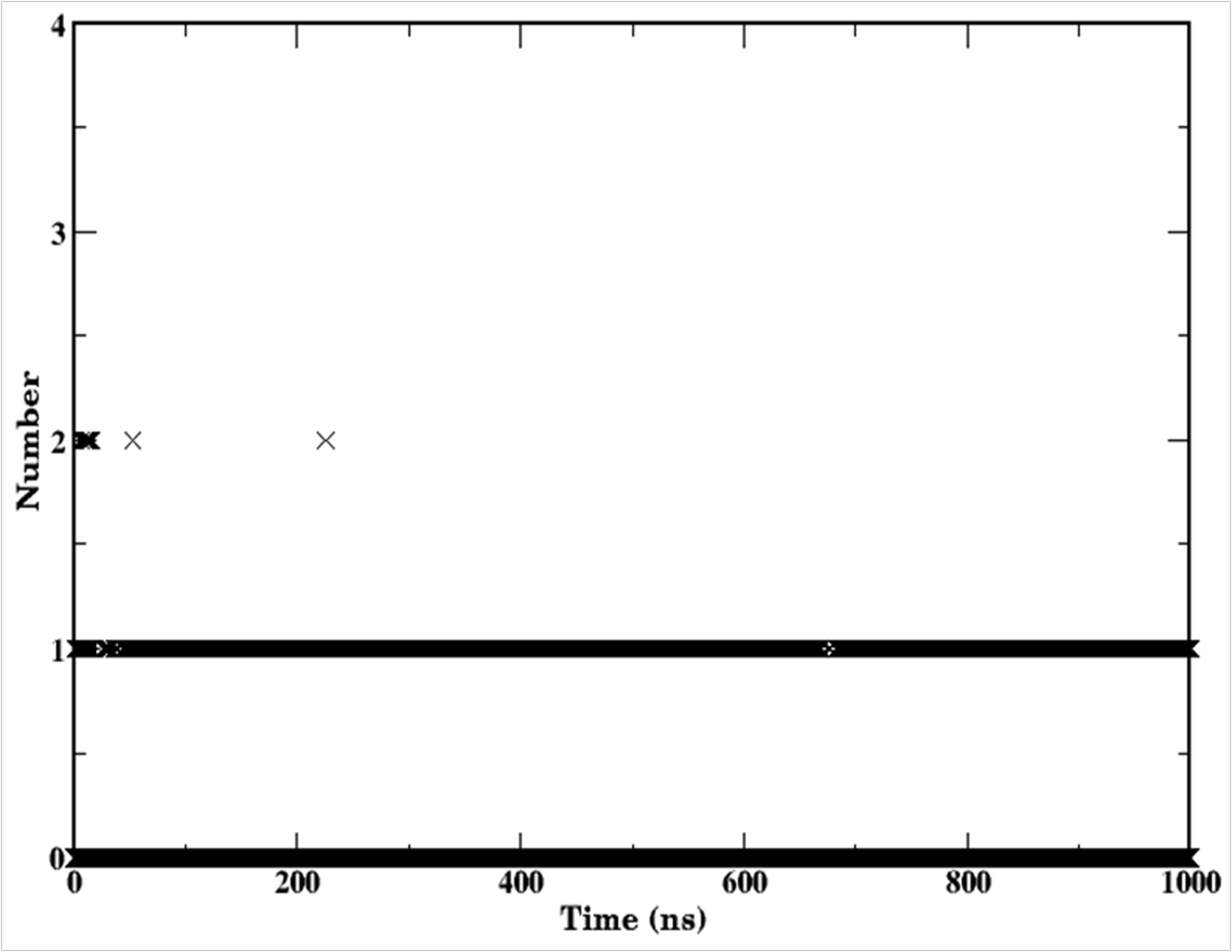

Supplement: Supplementary file 10 — Figure S6a. Profile of hydrogen bonds between Asp 21 on the N-terminal domain and Gln 443 on the TIR domain of TRIF along the MD trajectory. Figure S6b. Hydrogen bonds between Asp 21 and Leu 442 along the trajectory. Figure S6c. Hydrogen bonds between Lys 22 and Gln 471 over the MD trajectory. Figure S6d. Hydrogen bonds between Gln 20 and BB loop residue Glu 429. (ZIP 4528 kb) [file 13062_2017_179_MOESM10_ESM.zip › 10/Figure_S6cR2_600.tif]

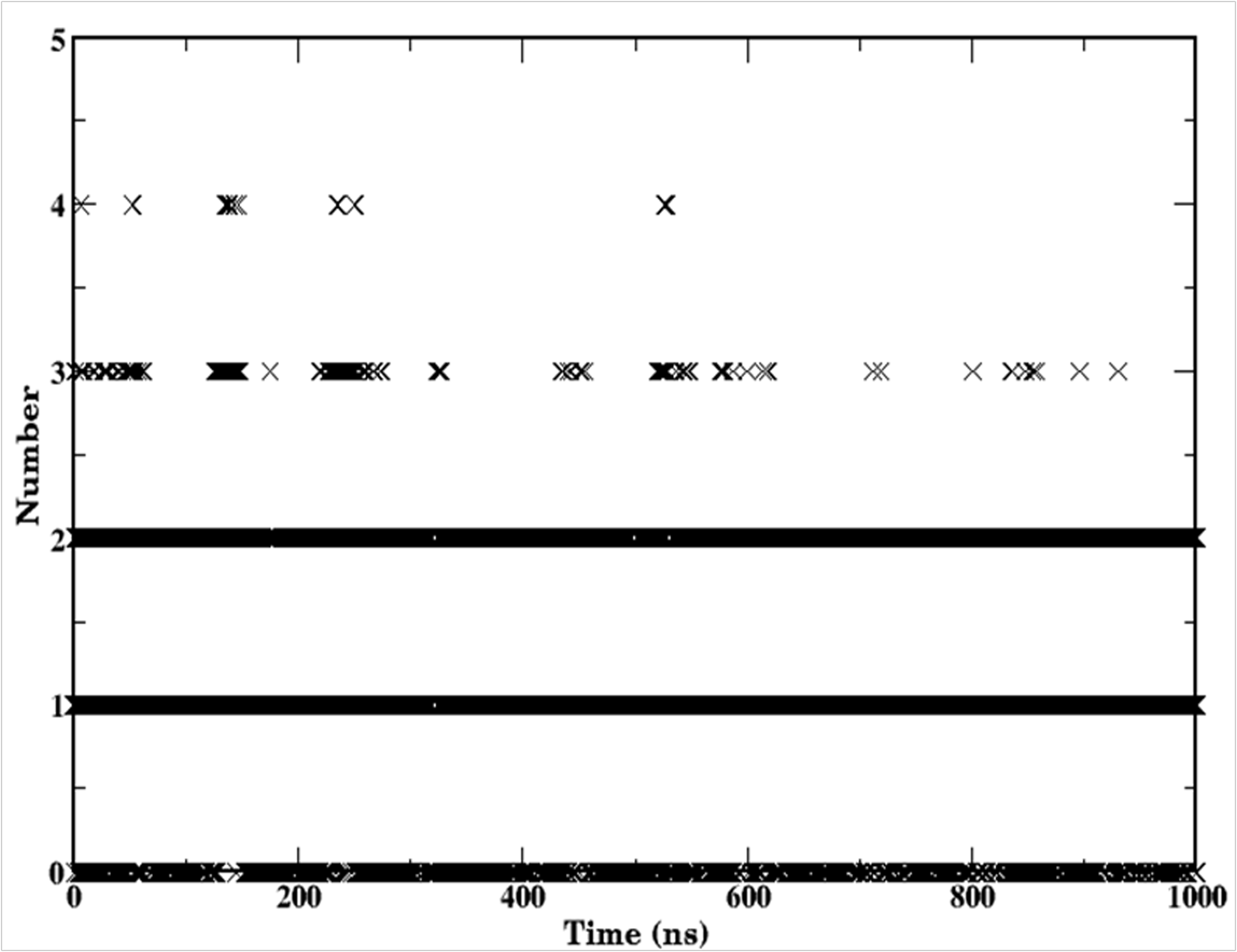

Supplement: Supplementary file 10 — Figure S6a. Profile of hydrogen bonds between Asp 21 on the N-terminal domain and Gln 443 on the TIR domain of TRIF along the MD trajectory. Figure S6b. Hydrogen bonds between Asp 21 and Leu 442 along the trajectory. Figure S6c. Hydrogen bonds between Lys 22 and Gln 471 over the MD trajectory. Figure S6d. Hydrogen bonds between Gln 20 and BB loop residue Glu 429. (ZIP 4528 kb) [file 13062_2017_179_MOESM10_ESM.zip › 10/Figure_S6dR2_600.tif]

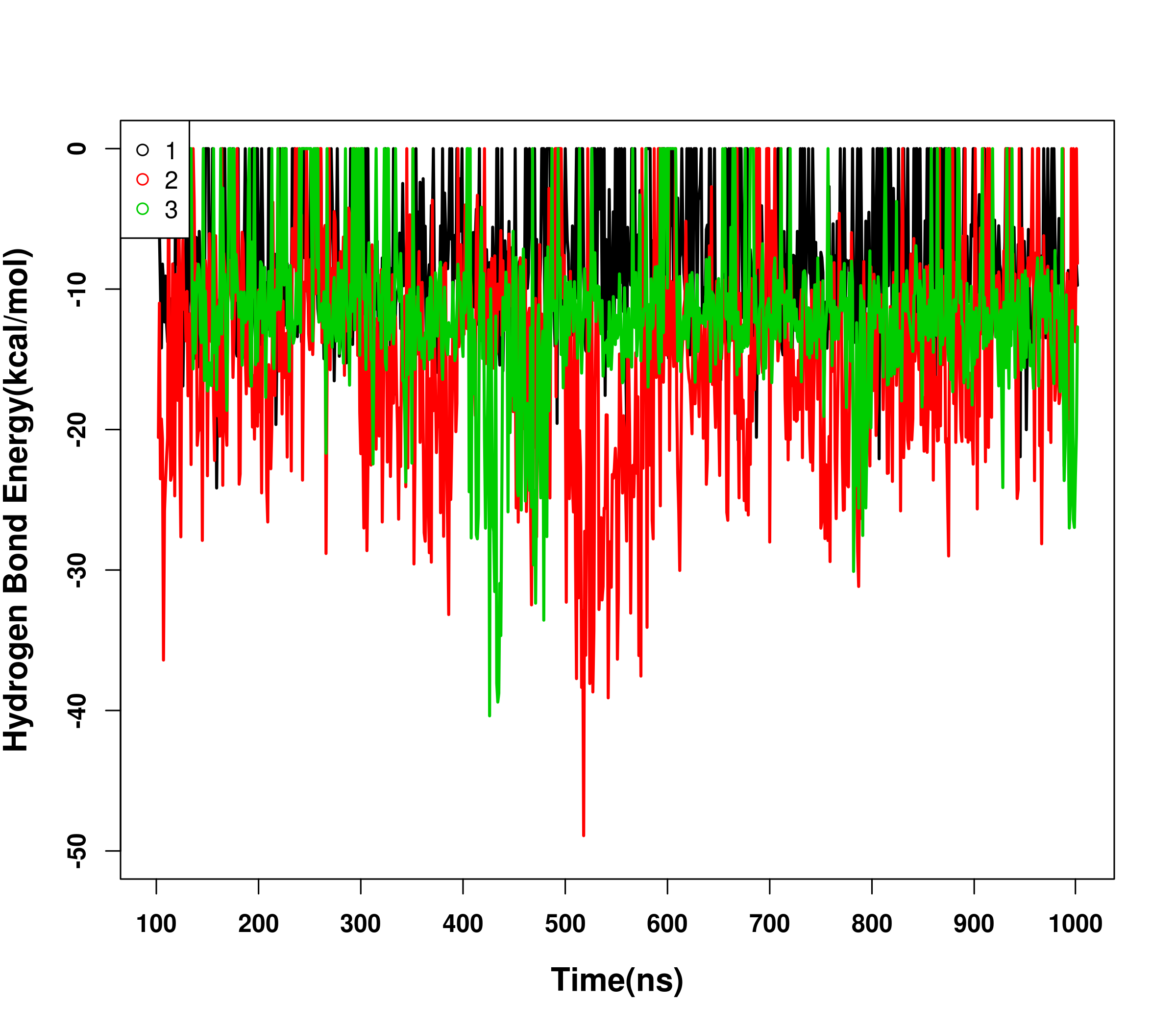

Supplement: Supplementary file 11 — Figure S7a. The hydrogen bond energy at the interface of the N-TIR complex calculated by PPCheck from different snapshots extracted every 1 ns from the trajectory, for all three replicates. Figure S7b. Variation in PPCheck-derived electrostatic energy at the interface during the course of the three MD simulations. Figure S7c:. van der Waals energies at the interface, calculated using PPCheck. Figure S7d. Total stabilizing energy at the interface. Figure S7e. Relative variation in the number of residues present at the interface. Figure S7f. Normalized energy per residue, as calculated using the PPCheck algorithm, at the interface. (ZIP 3290 kb) [file 13062_2017_179_MOESM11_ESM.zip › 11/Figure_S7aR2.tiff]

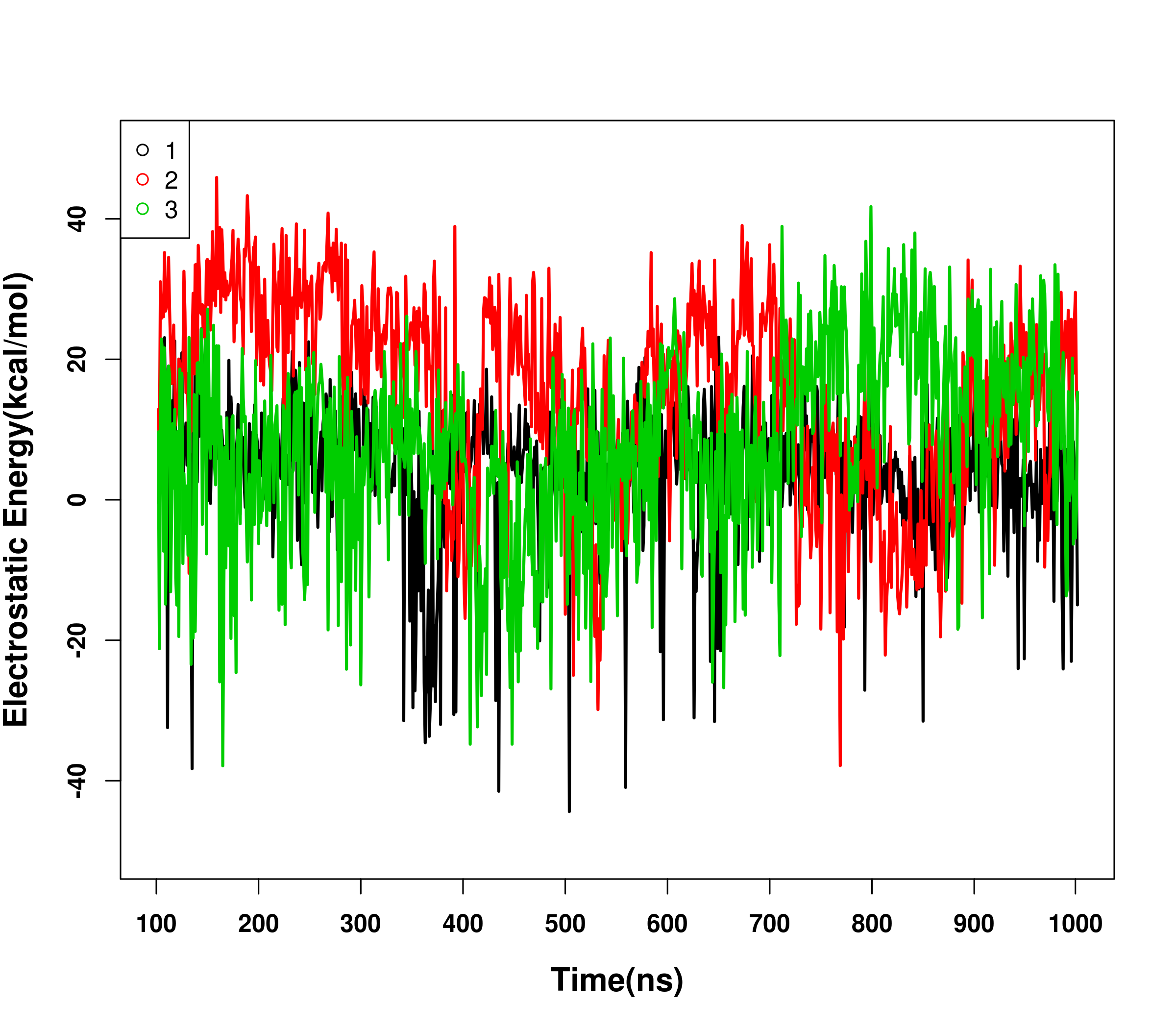

Supplement: Supplementary file 11 — Figure S7a. The hydrogen bond energy at the interface of the N-TIR complex calculated by PPCheck from different snapshots extracted every 1 ns from the trajectory, for all three replicates. Figure S7b. Variation in PPCheck-derived electrostatic energy at the interface during the course of the three MD simulations. Figure S7c:. van der Waals energies at the interface, calculated using PPCheck. Figure S7d. Total stabilizing energy at the interface. Figure S7e. Relative variation in the number of residues present at the interface. Figure S7f. Normalized energy per residue, as calculated using the PPCheck algorithm, at the interface. (ZIP 3290 kb) [file 13062_2017_179_MOESM11_ESM.zip › 11/Figure_S7bR2.tiff]

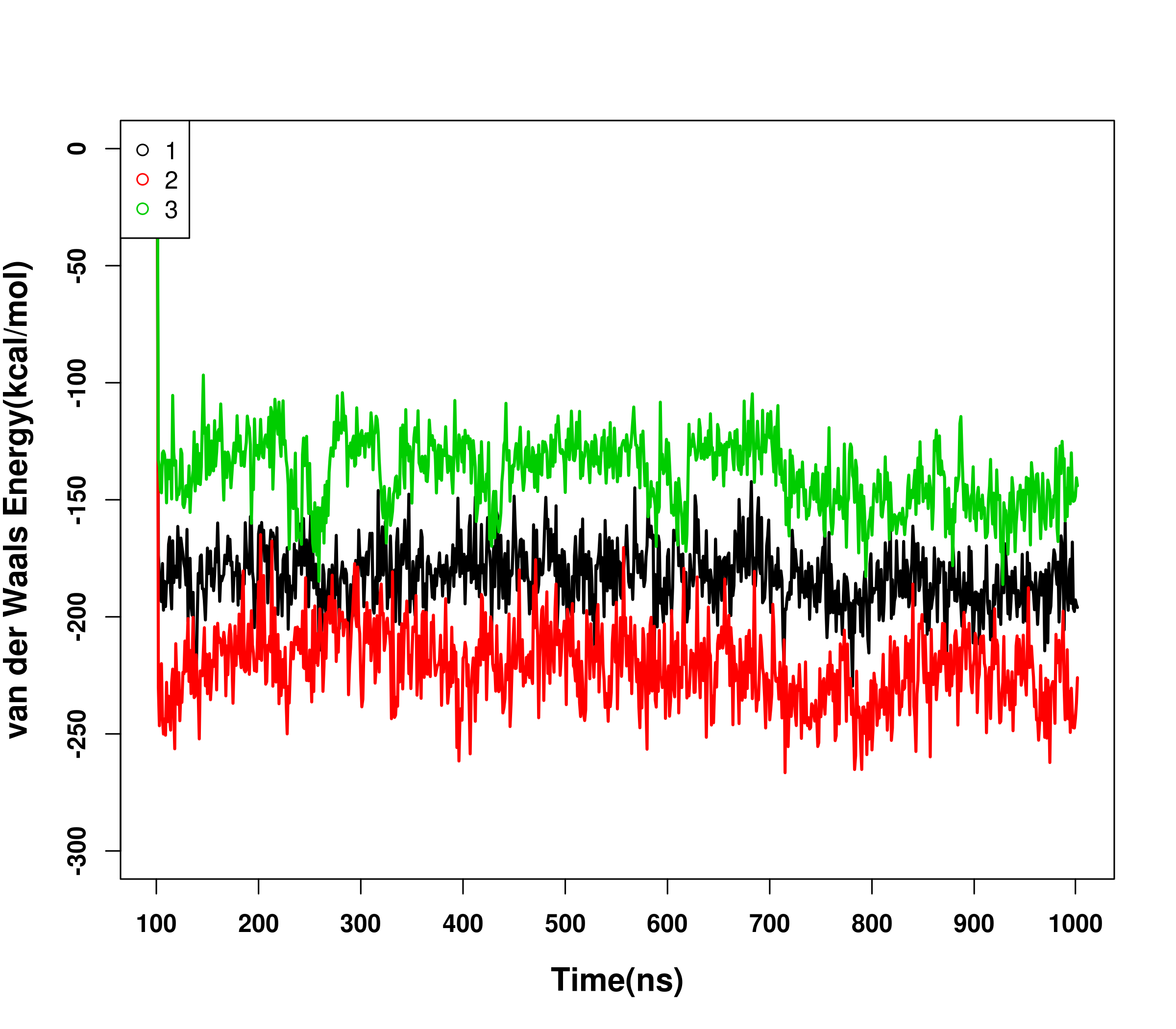

Supplement: Supplementary file 11 — Figure S7a. The hydrogen bond energy at the interface of the N-TIR complex calculated by PPCheck from different snapshots extracted every 1 ns from the trajectory, for all three replicates. Figure S7b. Variation in PPCheck-derived electrostatic energy at the interface during the course of the three MD simulations. Figure S7c:. van der Waals energies at the interface, calculated using PPCheck. Figure S7d. Total stabilizing energy at the interface. Figure S7e. Relative variation in the number of residues present at the interface. Figure S7f. Normalized energy per residue, as calculated using the PPCheck algorithm, at the interface. (ZIP 3290 kb) [file 13062_2017_179_MOESM11_ESM.zip › 11/Figure_S7cR2.tiff]

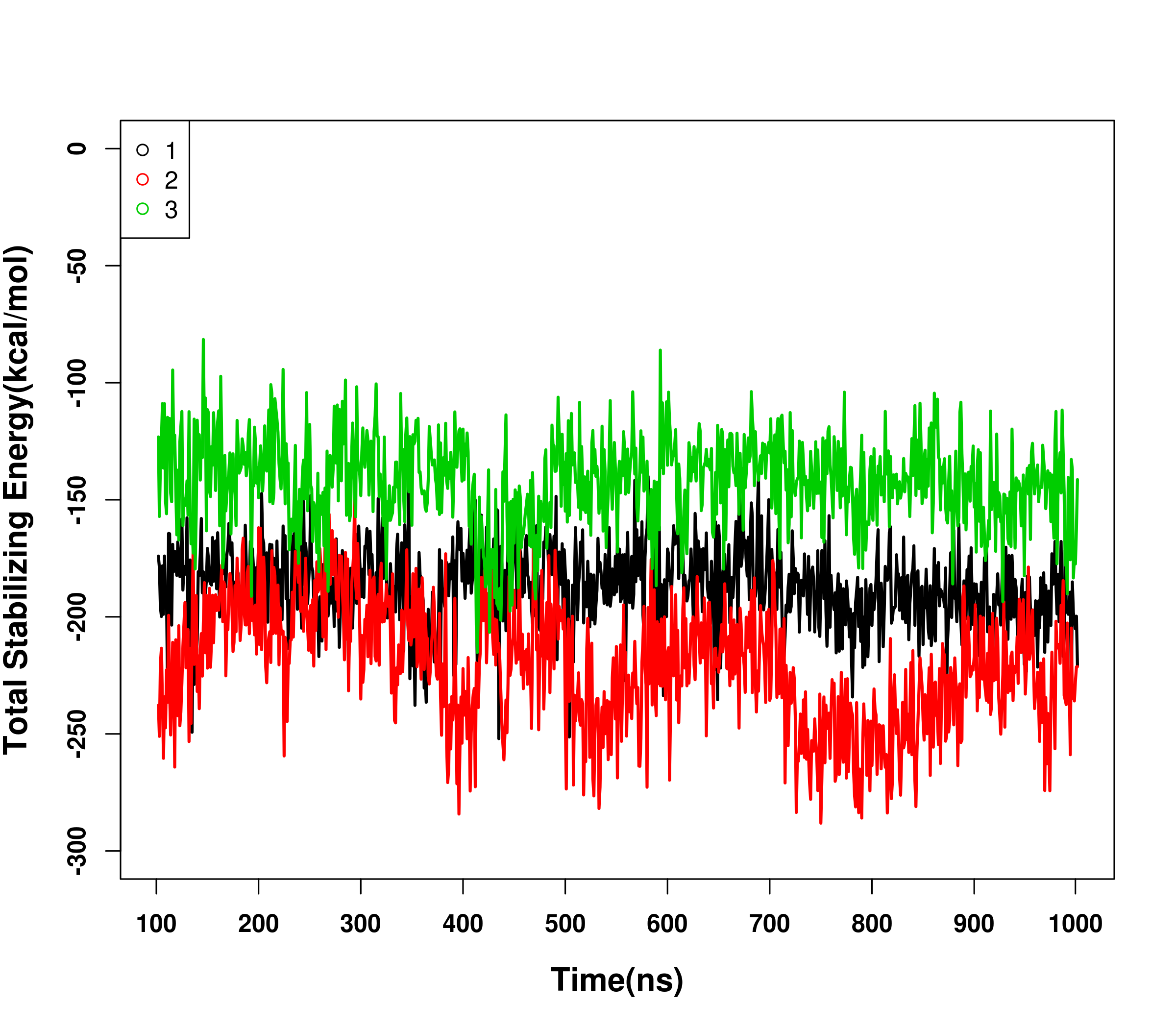

Supplement: Supplementary file 11 — Figure S7a. The hydrogen bond energy at the interface of the N-TIR complex calculated by PPCheck from different snapshots extracted every 1 ns from the trajectory, for all three replicates. Figure S7b. Variation in PPCheck-derived electrostatic energy at the interface during the course of the three MD simulations. Figure S7c:. van der Waals energies at the interface, calculated using PPCheck. Figure S7d. Total stabilizing energy at the interface. Figure S7e. Relative variation in the number of residues present at the interface. Figure S7f. Normalized energy per residue, as calculated using the PPCheck algorithm, at the interface. (ZIP 3290 kb) [file 13062_2017_179_MOESM11_ESM.zip › 11/Figure_S7dR2.tiff]

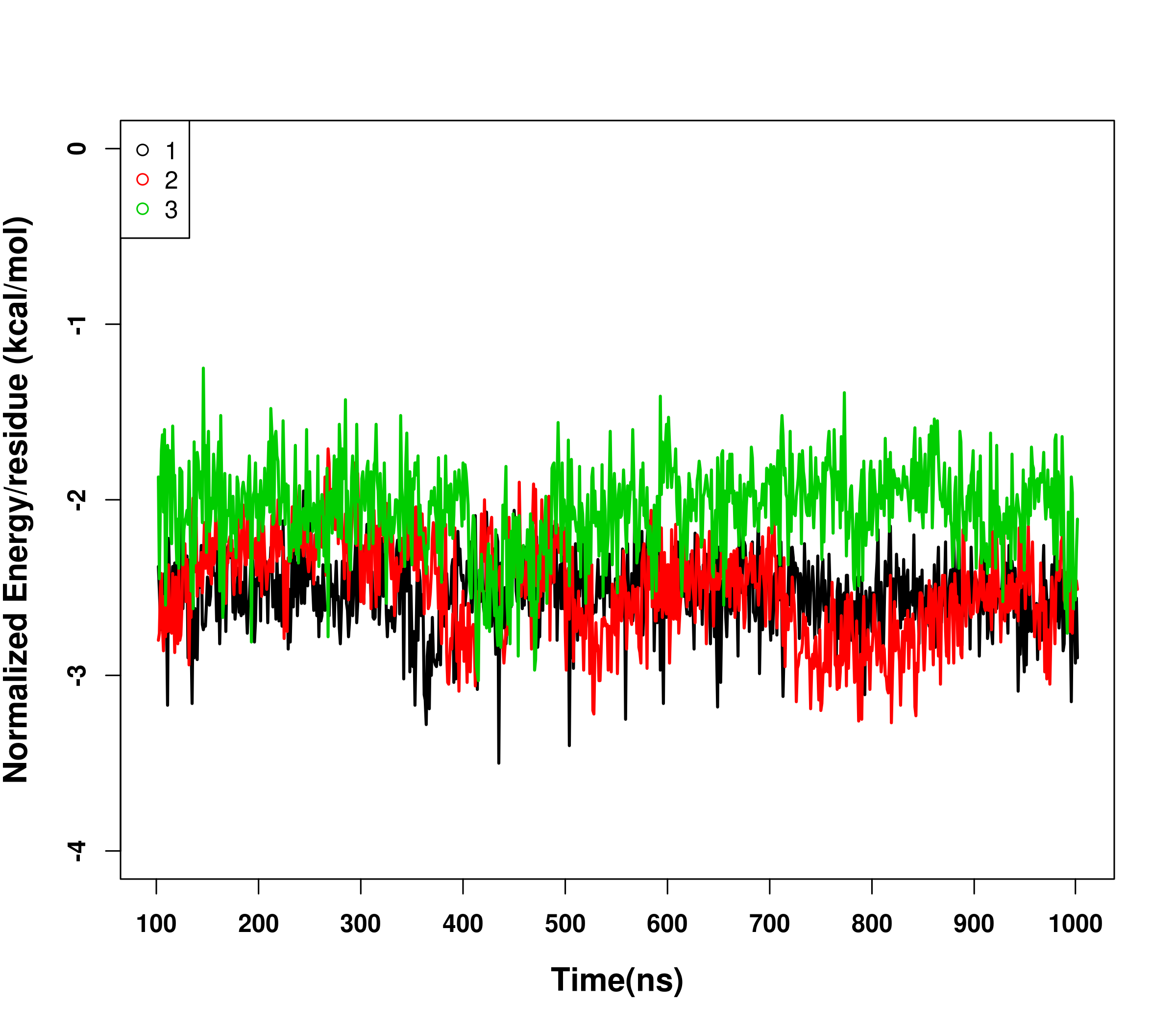

Supplement: Supplementary file 11 — Figure S7a. The hydrogen bond energy at the interface of the N-TIR complex calculated by PPCheck from different snapshots extracted every 1 ns from the trajectory, for all three replicates. Figure S7b. Variation in PPCheck-derived electrostatic energy at the interface during the course of the three MD simulations. Figure S7c:. van der Waals energies at the interface, calculated using PPCheck. Figure S7d. Total stabilizing energy at the interface. Figure S7e. Relative variation in the number of residues present at the interface. Figure S7f. Normalized energy per residue, as calculated using the PPCheck algorithm, at the interface. (ZIP 3290 kb) [file 13062_2017_179_MOESM11_ESM.zip › 11/Figure_S7eR2.tiff]

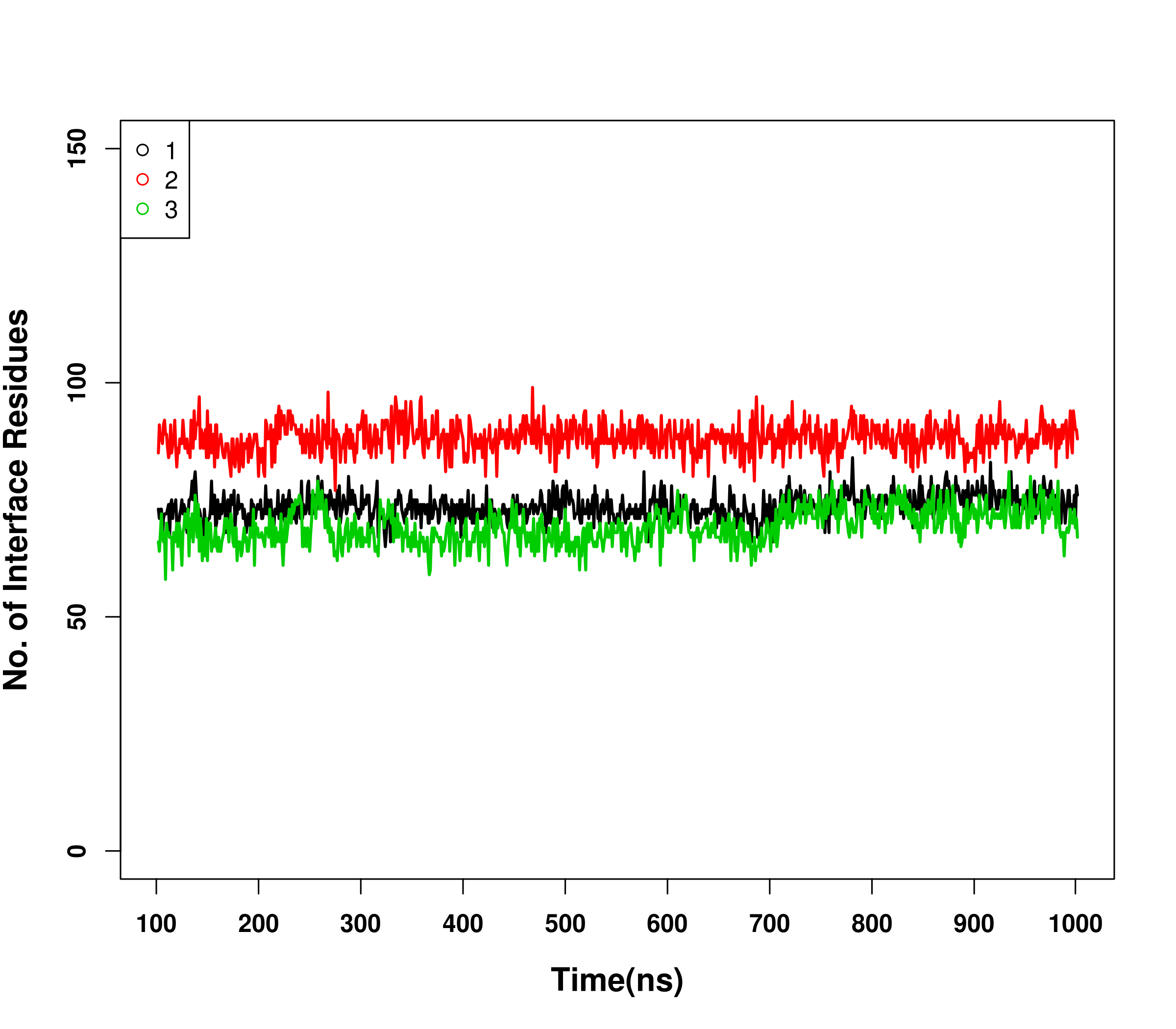

Supplement: Supplementary file 11 — Figure S7a. The hydrogen bond energy at the interface of the N-TIR complex calculated by PPCheck from different snapshots extracted every 1 ns from the trajectory, for all three replicates. Figure S7b. Variation in PPCheck-derived electrostatic energy at the interface during the course of the three MD simulations. Figure S7c:. van der Waals energies at the interface, calculated using PPCheck. Figure S7d. Total stabilizing energy at the interface. Figure S7e. Relative variation in the number of residues present at the interface. Figure S7f. Normalized energy per residue, as calculated using the PPCheck algorithm, at the interface. (ZIP 3290 kb) [file 13062_2017_179_MOESM11_ESM.zip › 11/Figure_S7fR2.tiff]

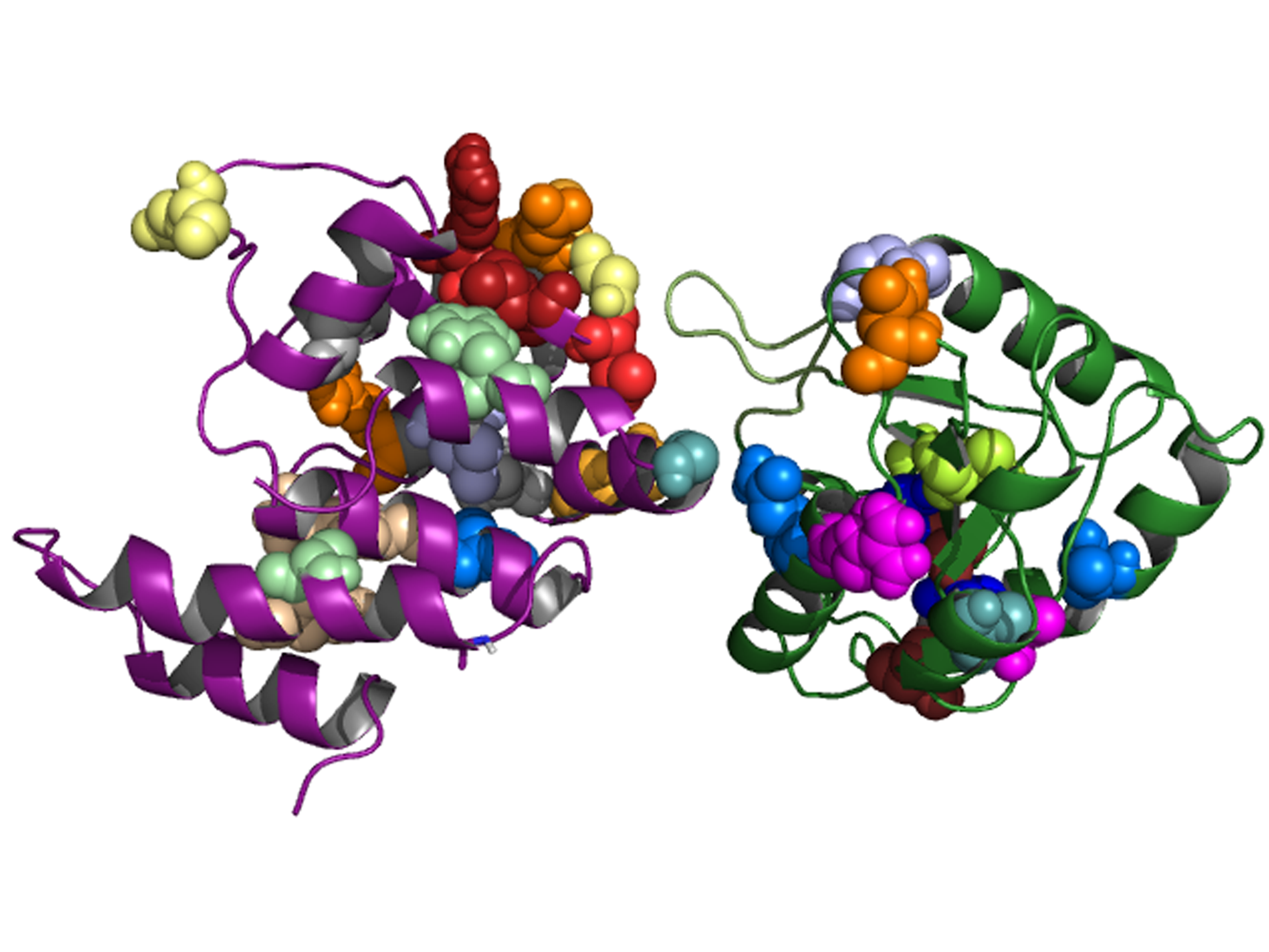

Supplement: Supplementary file 12 — Predicted co-evolving pairs of residues mapped onto the N-TIR docked complex. The colouring scheme follows the same scheme as in Additional file 3: Figure S1. (TIF 6339 kb) [file 13062_2017_179_MOESM12_ESM.tif]
