# Supplementary material for: Integrative modelling of TIR domain-containing adaptor molecule inducing interferon-β (TRIF) provides insights into its autoinhibited state
Source: Biol Direct. 2017 Apr 20;12:9. doi: 10.1186/s13062-017-0179-0 (PMC5397763; doi:10.1186/s13062-017-0179-0)
Supplement: Supplementary file 7 — Summary of interface residues identified in the different models generated by semi-guided docking using the Cluspro docking program. (DOCX 13 kb) [file 13062_2017_179_MOESM6_ESM.docx]

| **Residues used to guide the docking** | **Interactions identified by PPCheck in the top DOCKSCORE-ranked pose** | | | |
| --- | --- | --- | --- | --- |
|  | Number of Interface Residues | N-terminal domain interface residues participating in the interactions | TIR domain interface residues participating in the interactions | Nature of major interactions at the interface |
| Residues 428 to 439 on the BB loop of the TIR domain | 98 | Asp 21, Arg 34,Asp 67,Asp 91,Arg 98,Asp 130,Arg 132,His 131,Glu 139 | Glu 438, Glu 439,Asp 430,Arg 436,Gln 443,Asp 444,Asp 447, His 467,Gln 471 | Salt bridges,  Electrostatic |
| Residues comprising Region-1 of the N-terminal domain: Glu 40,Leu 49,Asp 67,Val 69,Glu 87,Glu 88,Asp 91, Trp 94,Leu 99,Arg 132,Glu 139 | 127 | Asp 21, Asp 41,Asp 67,Arg 71,Arg 98,Glu 139,Arg 141 | Glu 429,Arg 436,Asp 444,Arg 479,His 448, Gln 471,Glu 543 | Salt bridges |
| Residues comprising Region-2 of the N-terminal domain: Arg 98, Gln 119, Gln 120, Val 122, Gln 137, Asp 138, Glu 139, Arg 141, Gly 145 and Asp 147 | 110 | Arg 34,Asp 41,Asp 67,Arg 71, Arg 132, Glu 135,Asp 138,Glu 139 | Arg 403,Glu 429,Arg 463,Asp 461,His 467,Asp 502 | Salt bridges,  Electrostatic |
| Arg 522 and Lys 523 on the TIR domain | 87 | His 101, Glu 105,Lys 107,Ala 111, Arg 114,Asp 115,Glu 120,Arg 143,Asp 147 | Phe 394,Glu 391,His 448,Arg 479,Lys 542,Glu 543 | Salt bridges, Electrostatic |

**Table S2**:

Summary of interface residues identified in the different models generated by semi-guided docking using the Cluspro docking program.
