# Supplementary material for: Integrative modelling of TIR domain-containing adaptor molecule inducing interferon-β (TRIF) provides insights into its autoinhibited state
Source: Biol Direct. 2017 Apr 20;12:9. doi: 10.1186/s13062-017-0179-0 (PMC5397763; doi:10.1186/s13062-017-0179-0)

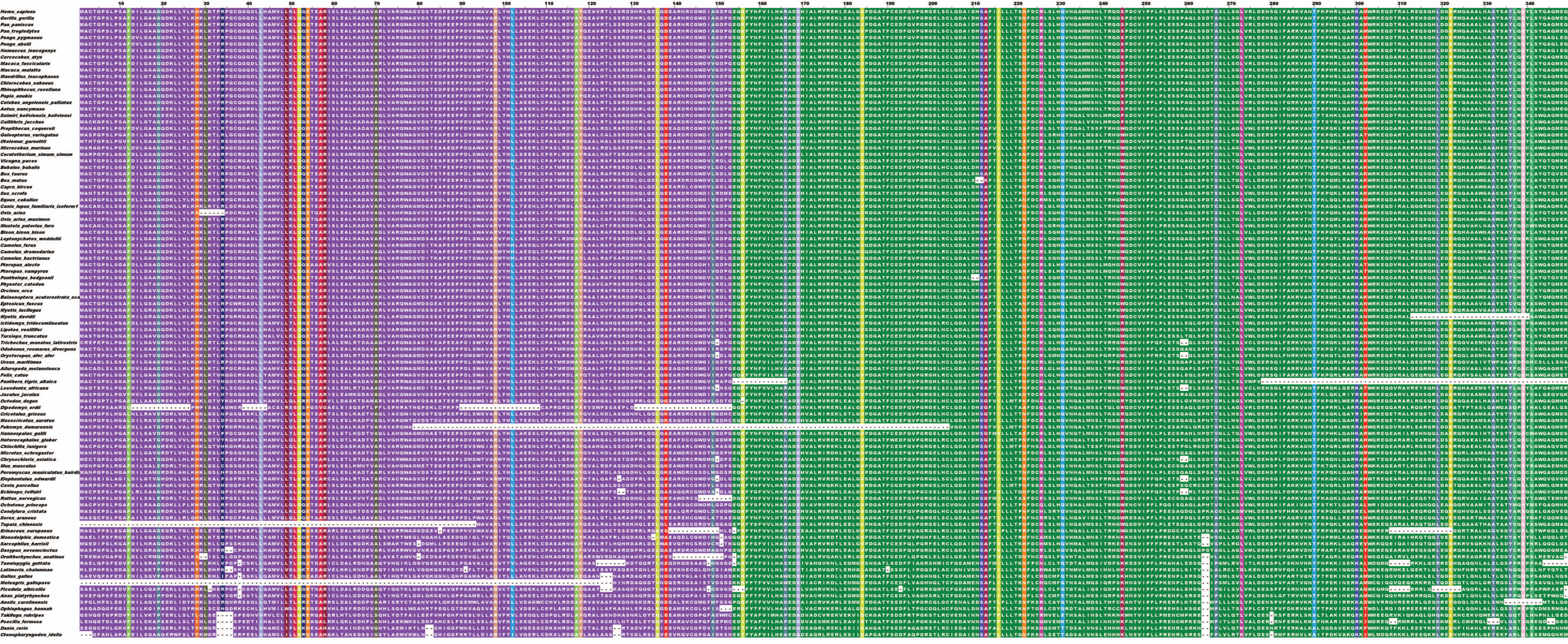

Supplement: Supplementary file 3 — The concatenated multiple sequence alignment of the N-terminal domain and TIR domain sequences from different organisms used for predicting co-evolving pair of residues. The region of the MSA corresponding to the N-terminal domain sequences is highlighted in purple while the region corresponding to the TIR domain is highlighted in green. Different colours for different co-evolving pair of residues with one colour for each pair have also been marked on the alignment. (PDF 4 MB) [file 13062_2017_179_MOESM2_ESM.pdf]
